# Impacts of high ATP supply from chloroplasts and mitochondria on the leaf metabolism of *Arabidopsis thaliana*

**DOI:** 10.3389/fpls.2015.00922

**Published:** 2015-10-28

**Authors:** Chao Liang, Youjun Zhang, Shifeng Cheng, Sonia Osorio, Yuzhe Sun, Alisdair R. Fernie, C. Y. M. Cheung, Boon L. Lim

**Affiliations:** ^1^School of Biological Sciences, The University of Hong KongPokfulam, Hong Kong; ^2^Max Planck Institute of Molecular Plant PhysiologyPotsdam-Golm, Germany; ^3^Departamento de Biología Molecular y Bioquímica, Instituto de Hortofruticultura Subtropical y Mediterranea, Universidad de Málaga-Consejo Superior de Investigaciones CientíficasMálaga, Spain; ^4^Department of Chemical and Biomolecular Engineering, National University of SingaporeSingapore, Singapore; ^5^State Key Laboratory of Agrobiotechnology, The Chinese University of Hong KongShatin, Hong Kong

**Keywords:** AtPAP2, ATP, chloroplasts, mitochondria, NADPH

## Abstract

Chloroplasts and mitochondria are the major ATP producing organelles in plant leaves. *Arabidopsis thaliana* purple acid phosphatase 2 (AtPAP2) is a phosphatase dually targeted to the outer membranes of both organelles and it plays a role in the import of selected nuclear-encoded proteins into these two organelles. Overexpression (OE) of AtPAP2 in *A. thaliana* accelerates plant growth and promotes flowering, seed yield, and biomass at maturity. Measurement of ADP/ATP/NADP^+^/NADPH contents in the leaves of 20-day-old OE and wild-type (WT) lines at the end of night and at 1 and 8 h following illumination in a 16/8 h photoperiod revealed that the ATP levels and ATP/NADPH ratios were significantly increased in the OE line at all three time points. The AtPAP2 OE line is therefore a good model to investigate the impact of high energy on the global molecular status of *Arabidopsis*. In this study, transcriptome, proteome, and metabolome profiles of the high ATP transgenic line were examined and compared with those of WT plants. A comparison of OE and WT at the end of the night provide valuable information on the impact of higher ATP output from mitochondria on plant physiology, as mitochondrial respiration is the major source of ATP in the dark in leaves. Similarly, comparison of OE and WT following illumination will provide information on the impact of higher energy output from chloroplasts on plant physiology. OE of AtPAP2 was found to significantly affect the transcript and protein abundances of genes encoded by the two organellar genomes. For example, the protein abundances of many ribosomal proteins encoded by the chloroplast genome were higher in the AtPAP2 OE line under both light and dark conditions, while the protein abundances of multiple components of the photosynthetic complexes were lower. RNA-seq data also showed that the transcription of the mitochondrial genome is greatly affected by the availability of energy. These data reflect that the transcription and translation of organellar genomes are tightly coupled with the energy status. This study thus provides comprehensive information on the impact of high ATP level on plant physiology, from organellar biology to primary and secondary metabolism.

## Introduction

Energy is the driving force of growth and manifold biological processes are regulated by its availability. In plants, photosynthesis is the ultimate source of energy and reducing power. Under illumination, photosystems transform light energy into ATP and NADPH, which in turn are used to fix CO_2_ by the Calvin–Benson cycle. Carbon fixed during the daytime is mainly stored as starch in chloroplasts or exported to the cytosol for sucrose synthesis. During the night time when photosynthetic ATP generation does not occur, key catabolic processes like glycolysis in the cytosol and the plastid, and the tricarboxylic acid (TCA) cycle and oxidative phosphorylation in mitochondria break down carbohydrates to generate ATP. Hence, the two powerhouses of plant cells, chloroplasts and mitochondria, coordinate to generate adequate ATP in plants to meet energy demands of various anabolic processes (Khlyntseva et al., 2009). ATP is not solely consumed for biosynthesis, but also in transport of metabolites, proteins and RNA, and turnover of macromolecules such as proteins, RNA and DNA. For example, it was estimated that ∼1000 ATP molecules are consumed for the import of one protein molecule across membrane (Alder and Theg, 2003). Hence, biomass synthesis only contributes to a small proportion of the total energy requirement in plant cells (Masakapalli et al., 2010).

Reducing power generated from photosynthesis is also a driving force of many biological processes. Through photosynthetic linear electron flow (LEF), electrons from water molecules are transferred to ferridoxin (Fd). Electrons in reduced Fd can be passed to NADP^+^ to generate NADPH or to thioredoxins (TXs). Reduced TXs, in turn, reduce and activate several key enzymes in the Calvin–Benson cycle (Oelze et al., 2008; Foyer et al., 2012), as well as in the processes of nitrogen (nitrite reductase, NiR) and sulfur (sulfite reductase, SR) assimilation (Meyer et al., 2005; Oelze et al., 2008). Excess reducing power generated from photosynthesis can be exported from chloroplasts in the form of malate, which can thereby provide reducing power (in the form of NADH) to other organelles (Scheibe, 2004; Noguchi and Yoshida, 2008), for example, to the mitochondria for ATP production through the respiratory electron transport chain (Bailleul et al., 2015). As is the case for ATP, it has been suggested that a substantial amount of reducing power is consumed for cellular maintenance (Cheung et al., 2013).

Since energy supply has such a great impact on plant physiology, it is of considerable value to understand how the physiology of plant cells is affected by the availability of energy. Here we use tools of experimental systems biology to characterize a previously well characterized high ATP containing fast-growing, transgenic *Arabidopsis* line that overexpresses *Arabidopsis thaliana* purple acid phosphatase 2 (AtPAP2; Zhang et al., 2012) and compare these characteristics to those of wild-type (WT) plants. The AtPAP2 overexpression (OE) lines flower earlier and grow faster than the WT lines, and the silique number and seed yield of the transgenics exceed those of WT plants (Zhang et al., 2012). Similarly, OE of AtPAP2 in *Camelina sativa* and potatoes also resulted in higher yield and fast growth (Zhang et al., 2012, 2014). Microarray studies additionally revealed that thousands of transcripts were altered in the transgenic *Arabidopsis* lines (Sun et al., 2013). When taken together these phenotypes suggest a dramatic shift of metabolism in the AtPAP2 OE lines.

Purple acid phosphatases (PAPs) are a set of metalloenzymes that hydrolyze phosphoesters in acidic conditions (pH 4-7) (Klabunde et al., 1996). Twenty-nine PAP genes were identified in the genome of *A. thaliana* (Li et al., 2002) but only two members carry a C-terminal hydrophobic motif (AtPAP2 and AtPAP9). The C-terminal hydrophobic motif of AtPAP2 anchors it to the outer membranes of chloroplasts and mitochondria and the OE of AtPAP2 on these two powerhouses modify the physiology of chloroplasts and mitochondria by regulating the import process of selected proteins into these two organelles (Law et al., 2015; Zhang et al., unpublished).

In this study we found that the OE line contains significantly higher level of ATP than the WT at the end of night in a 16/8 h photoperiod and at 1 and 8 h following the incidence of irradiation. A comparison of OE and WT at the end of the night can provide valuable information on the impact of higher ATP output from mitochondria on plant physiology, as mitochondrial respiration is the major source of ATP in the dark in leaves. Similarly, comparison of OE and WT at 1 and 8 h following illumination will provide information on the impact of higher energy output from chloroplasts on plant physiology. Since many components of the energy generating systems (the photosynthetic and the respiratory electron transport chains in the chloroplast and the mitochondria, respectively) are encoded by both the nuclear and organellar genomes, the transcription data of organellar genomes also present important information. Here, whole genome transcriptomic data, including transcripts expressed in the mitochondrial and chloroplast genomes, was studied by RNA-seq after removal of ribosomal RNA. Moreover, proteomics of leaves was compared between the well-characterized OE line and WT at the end-of-night and the middle-of-day, which provided information on translational regulation under the influence of energy. The difference in mRNA and protein levels of key processes and metabolic pathways such as the photosynthetic light reactions, the Calvin–Benson cycle, the TCA cycle, redox reactions, glycolysis, and oxidative phosphorylation were studied in detail. These combined data thus allowed us to provide a comprehensive systems-based analysis of the impact of ATP level on leaves in the light or dark. The results are discussed in the context of current models of the regulation of energy-associated metabolism.

## Materials and Methods

### Plant Materials, Growth Conditions, and Experimental Design

*Arabidopsis thaliana* ecotypes Columbia (Col-0) (WT), AtPAP2 overexpressors (OE in Col-0 background) were used in this study (Zhang et al., 2012). The seeds were placed on Murashige and Skoog medium supplemented with 2% (w/v) sucrose for 10 days, then the seedlings were transferred to soil under 16 h light (22°C)/8 h dark (18°C) period in growth chamber at a light intensity of 120–150 μmol m^-2^ s^-1^. The rosette leaves of 20-day-old *Arabidopsis* before bolting were harvested at three different time points: *t* = 0 h (end of night), *t* = 1 h (one hour after onset of illumination), and *t* = 8 h (eight hours after onset of illumination) and frozen in liquid nitrogen before RNA, protein and metabolite extraction. Plants were pooled for each biological replicate. The same batch of samples was used for transcriptomic, proteomic, and metabolomic studies.

### ATP/ADP/NADP(H) Extraction and Measurement

ADP and ATP were extracted from leaves by a trichloroacetic acid method. The level of ATP can be directly measured by the Bioluminescent Assay Kit (Sigma, FL-AA) (Ford and Leach, 1998). To measure the level of ADP, ADP in the extract was first converted into ATP by pyruvate kinase (PK) and the sum was measured (Meyer et al., 2009).

The extraction of nicotinamide adenine dinucleotide phosphates (NADP^+^ and NADPH) was based on the selective hydrolysis of NADPH in acid medium and selective hydrolysis of NADP^+^ in alkaline medium (Foyer et al., 1991). The neutralized mixture was incubated on ice for 15 min, before being frozen in liquid nitrogen and stored in –80°C (Hajirezaei et al., 2002). After pH adjustment, the levels of NADP^+^ and NADPH were measured in 96-well plates according to a plate reader method (Queval and Noctor, 2007). Standard curves of 0–40 pmol authentic standard were freshly prepared on the day of measurement.

### Metabolomics Analysis

Metabolite profiling of *Arabidopsis* leaves by gas chromatography–mass spectrometry (GC–MS) (ChromaTOF software, Pegasus driver 1.61; LECO) as described previously (Lisec et al., 2006). The chromatograms and mass spectra were evaluated using TagFinder software (Luedemann et al., 2012). Metabolite identification was manually supervised using the mass spectral and retention index collection of the Golm Metabolome Database (Kopka et al., 2005). Peak heights of the mass fragments were normalized on the basis of the fresh weight of the sample and the added amount of an internal standard (ribitol).

### Transcriptome Analysis

Total RNA was extracted from leaves at all three time points and DNA contamination was removed by DNase I (RNeasy Plant Mini Kit, Qiagen, Hong Kong). The preliminary quality was detected by running 1% (w/v) agarose gel stained by GelRed (Biotium, USA). The quality of RNA samples was tested with Agilent 2100 Bioanalyzer RNA Nanochip. At least 20 μg of total RNA at a concentration of ≥300 ng/μl, OD260/280 ≥ 1.8, OD260/230 ≥ 1.8, RNA 28S:18S ≥ 1.2, RIN ≥ 6.5 were used for cDNA library preparation. Ribosomal RNAs were removed from the total RNA by the Ribo-Zero rRNA removal kit for plant leaf (Epicentre, USA) before cDNA library construction. Plants were collected and pooled together for RNA extraction. Each pooled RNA was considered as one biological replicate. The libraries were sequenced using Illumina HiSeq^TM^2000. After removal of low quality reads, clean reads from three different RNA-seq samples (both WT and OE, 3 times points, *t* = 0, 1, 8 h) were aligned to *Arabidopsis thaliana* genome (TAIR 10.0). All the raw data were deposited in to NCBI GEO^1^ with accession number GSE57790 and GSE57791.

To distinguish the homologous transcripts derived from nucleus and organelles, the clean reads were mapped to the *Arabidopsis thaliana* genome (TAIR10) with nucleus-encoded CDS gene set, the mitochondria-encoded CDS gene set, and the chloroplast-encoded CDS gene set. SOAPaligner/SOAP2 was used as the alignment tool with the following parameters: -m 0 -x 10000 -s 40 -l 32 -v 5 -r 2 -p 6 (Li et al., 2009). The transcript abundance was estimated by RPKM (Reads per kilobase transcript per million reads) calculation for each gene in each compartment (Mortazavi et al., 2008) by the equation RPKM = 10^9∗^*C*/*N*^∗^*L*, where *C* is the number of mappable reads that fell onto the genes, *N* is the total number of mappable reads in the experiment, and *L* is the gene length.

In order to filter the differentially expressed transcripts between sapmles, all reads were sequenced and *p*-value was calculated according to a strict algorithm (Audic and Claverie, 1997). The reads from gene *A* is denoted as *x*, and when the expression of each given transcripts occupies only a small part of the library, *x* yields to the Poisson distribution:

$$p\left(x\right)=\frac{e^{-\lambda }\lambda^{x}}{x!}\left(\lambda isthereadtranscriptsofthegene\right)$$

The total clean tag number of WT is *N*_1_, and total clean tag number of OE is *N*_2_; gene *A* holds *x* tags in WT and *y* tag in OE. Therefore, the probability of gene *A* expressed equally between the two compared samples can be calculated by:

$$2\;\;\overset{i=y}{\underset{i=0}{\Sigma }}\;p\left(i|x\right)$$

or

$$2\times (1-\overset{i-y}{\underset{i=0}{\Sigma }}p\left(i|x\right))\left(if\overset{i-y}{\underset{i=0}{\Sigma }}p\left(i|x\right)>0.5\right)$$

$$\;p\left(y|x\right)={\left(\frac{N_{2}}{N_{1}}\right)}^{y}\frac{(x+y)!}{x!y!{(1+\frac{N_{2}}{N_{1}})}^{\left(x+y+1\right)}}$$

Besides the *p*-value calculation, correction for false positive (type I errors) and false negative (type II) errors were performed using FDR method (Benjamini et al., 2001). Finally, transcripts that have FDR ≤ 0.001 and fold change >2 were considered as differentially expressed.

The PageMan software package (Usadel et al., 2006) was used to select and display biologically relevant details of these data sets.

### Leaf Protein Extraction, iTRAQ Labeling, and LC–MS/MS

Proteins were extracted by the trichloroacetic acid/acetone method. The protein pellet was dissolved with 2 ml urea buffer (6 M Urea and 4 mM CaCl_2_ in 200 mM MOPS, pH 8.0) (Ross et al., 2004). Equal amount of proteins (100 μg) were reduced by 10 mM dithiothreitol (DTT) and alkylated by 40 mM iodoacetamide (IAA) in the dark. After alkylation, the mixture was diluted with 4 mM CaCl_2_ to reduce the concentration of urea to less than 2 M. Trypsin was added to digest protein at 1:20 ratio at 37°C overnight. Following trypsin digestion, the peptides were desalted by C18 SepPak reverse-phase cartridges (Waters, WAT023590, and Ireland). The desalted peptides were then labeled by the 8-plex isobaric tags for relative and absolute quantitation (iTRAQ) labeling kit (AB Sciex, USA). Two biological replicates of each sample were used for iTRAQ labeling. WT (*t* = 0 h), OE (*t* = 0 h), WT (*t* = 8 h), OE (t = 8 h) were labeled with 113, 114, 115, and 116, respectively. Then the second biological replicate samples were labeled with 117, 118, 119, and 121, respectively. All labeled samples (eight tubes) were combined together and the labeled peptides were fractionated by SCX (Zhao et al., 2012). Fractions collected from 15 to 40 mins were combined into 12 fractions for LC/MS/MS analysis (TripleTOF 5600 system, AB SCIEX, USA). Two technical replicates were run. MS data were acquired using a TripleTOF 5600 system fitted with a Nanospray III source (AB SCIEX, USA) (Siu et al., 2011).

### Proteomics Analysis

Tandem mass spectrometry (MS/MS) data was analyzed using the Paragon algorithm in ProteinPilot 4.0 software (Applied Biosystems, USA). The raw data obtained from the machine were converted to.mgf from.wiff format file by PeakView software. To qualify and quantify the protein abundance changes of the nucleus- and organelle-encoded genes under different conditions, we searched the protein IDs and peptides mapped by ProteinPilot software against the *Arabidopsis* nucleus-encoded protein database, mitochondria-encoded protein database, and chloroplast-encoded protein database from the TAIR website^2^. Downstream analysis for the calculation of the protein expressed level for each gene from each compartment was conducted by a series of in-house perl scripts. In all searches, trypsin was selected as the enzyme used for protein digestion and IAA was selected as the cysteine alkylation agent. Bias correction and background correction were also applied. For protein identifications, a minimal unused ProtScore of 1.3 with at least two peptides (confidence ≥ 95%) was necessary. The false discovery rate (FDR) analysis was performed using the PSPEP add-on function of ProteinPilot based on a decoy database of reverse sequences. All four replicated ratios were used for statistical analysis by using one sample *t*-test (one-tailed test) using the following formula: $t=\frac{x-\mu 0}{s/\sqrt{n}},$, where *x* is the mean ratio of the four replicates, μ_0_ is the assumed value (we assumed three values 1.2, 1.33, and 1.5, respectively), *s* is the standard deviation of the four replicates and *n* is the number of the replicates. The degree of freedom used was 3 and *P* < 0.05 was regarded as statistically significant.

### Quantitative Reverse Transcriptase-PCR

Quantitative reverse transcriptase PCR (qRT-PCR) analysis was carried out using cDNA samples transcribed from the same batch of RNA used in transcriptome. Primer premier 5.0^3^ was used to design the qRT-PCR primers (**Supplementary Table S1**). The PCR reactions were performed in a 10 μL volume containing a 2× SYBR Green Master Mix (ABI systems). The amplification parameters were 95°C for 1 min; followed by 40 cycles of 15 s of 95°C and 1 min of 60°C. Actin 2 (*At3g18780*) was used as the internal control. For every transcript, each cDNA sample was analyzed in triplicate, and relative transcript abundance was calculated by normalizing to the maximum level. The assessment of expression comparing different targets was determined by the ddCt comparative threshold (ΔΔCt) method. *P*-values were determined by a two-tailed paired Student’s *t-*test.

## Results

### Impact of AtPAP2 OE on Leaf ADP, ATP, NADP^+^, and NADPH Levels

**FIGURE 1:**
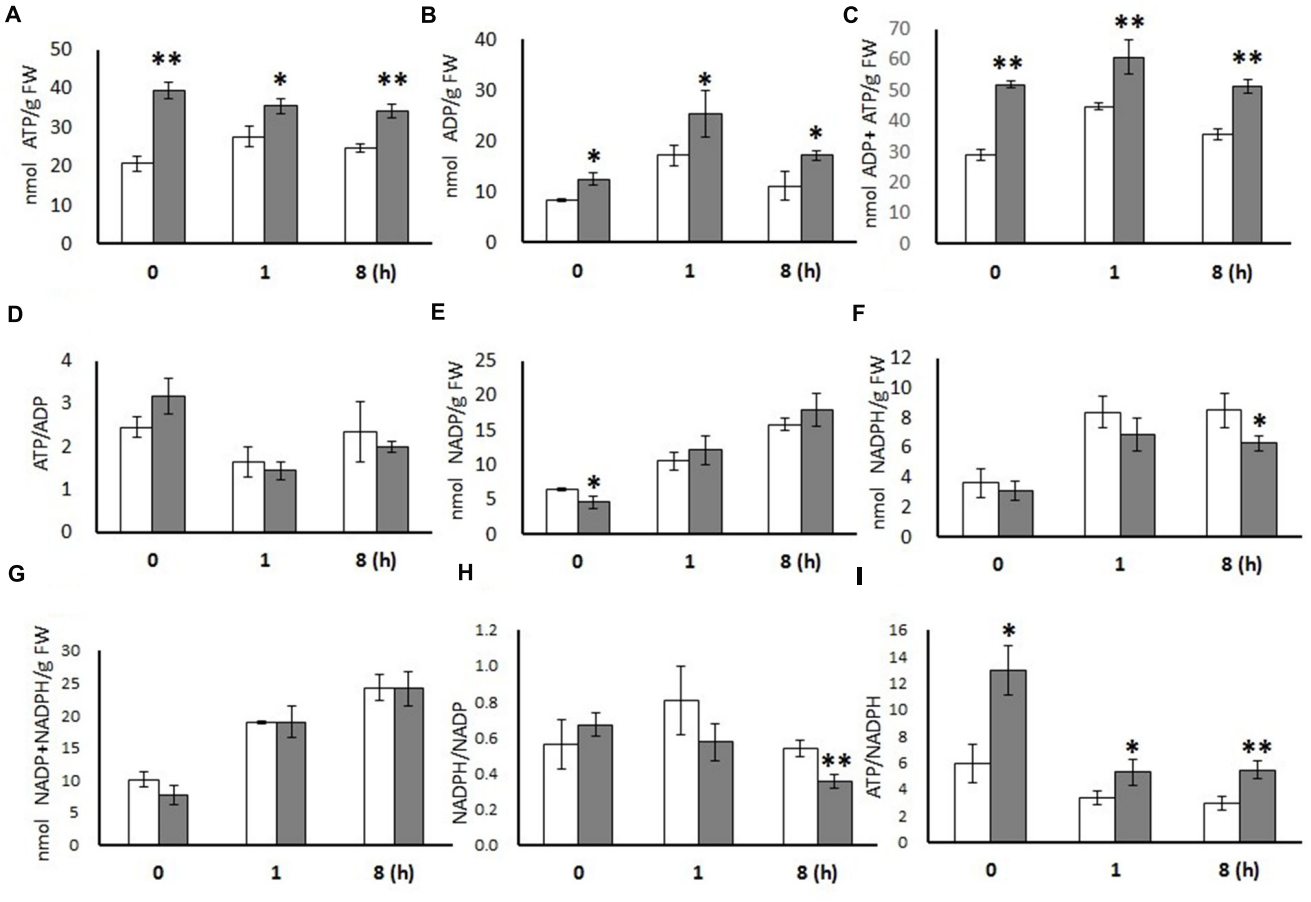
**Metabolites measured from 20-days-old leaves of WT and OE7 *Arabidopsis* at different time points.** ATP **(A)**, ADP **(B)**, ADP + ATP **(C)**, ATP/ADP **(D)**, NADP **(E)**, NADPH **(F)**, NADP + NADPH **(G)**, NADPH/NADP **(H)**, and ATP/NADPH **(I)**. Data are expressed (nmol/g FW) as means with ±SD of three biological replicates. Independent sample *t*-test using IBM SPSS Statistics 19 Software. Asterisks indicate significant difference between WT (white bar) and OE (gray bar), ^∗^*P* < 0.05, ^∗∗^*P* < 0.01. FW, Fresh weight.

ADP and ATP levels were significantly higher in OE leaves than in WT leaves at all three time points (*t* = 0, 1, and 8 h), while the ratios of ATP/ADP were not statistically changed (**Figure 1**). NADP^+^ content was significantly lower in the OE line only in dark conditions. NADPH content was significantly lower in OE leaves in the middle of light period (*t* = 8 h) and as a result, the deduced NADPH/NADP^+^ ratio was lower. Similarly, the ATP/NADPH ratios were considerable higher in the OE line at all time points (*t* = 0, 1, and 8 h), which was mainly a consequence of the higher ATP levels.

### RNA-seq Data Analysis Identifies Multiple Differentially Expressed Transcripts

Total sequenced reads were mapped both to *Arabidopsis* TAIR 10 genes (**Supplementary Table S2A**) and to the genome (**Supplementary Table S2B**). In total, 29,500 expressed transcripts were detected in the RNA-seq data, including 29,278 transcripts encoded by the nuclear genome, 126 transcripts encoded by the mitochondrial genome, and 96 transcripts encoded by the chloroplast genome (**Supplementary Tables S3**–**S5**). The 29,278 nuclear transcripts were encoded by a total of 23,250 genes, where the difference between these two numbers is due to the fact that some genes display alternative splicing. RNAs were classified as protein coding, pre-tRNA and rRNA, respectively. The gene type destinations were compared with those published in TAIR 10 *Arabidopsis* Columbia-0 (Col-0)^4^ (**Table 1**).

**Table 1:** Gene type destinations mapped to TAIR 10.0 genome database.

	Total	Protein coding	pre-tRNA	rRNA	snRNA	snoRNA	miRNA	Other RNA	Pseudogene	TE
Chr1-5	23,250	21,497/27206	0/631	2/4	13/13	18/71	66/177	339/394	368/924	947/3903
ATMG	126	121/122	2/21	3/3	0	0	0	0	0	0
ATCG	96	87/88	1/37	8/8	0	0	0	0	0	0
Total expressed genes	23,472	21,705/27416	3/689	13/15	13/13	18/71	66/177	339/394	368/924	947/3903


*Numbers in denominator are the total gene number of each type of RNAs annotated in TAIR 10.0 database (http://www.*arabidopsis*.org/portals/genAnnotation/genome_snapshot.jsp). Chr1-5, ATMG, and ATCG represent chromosomes 1–5, mitochondrial and chloroplast genomes, respectively.*

**FIGURE 2:**
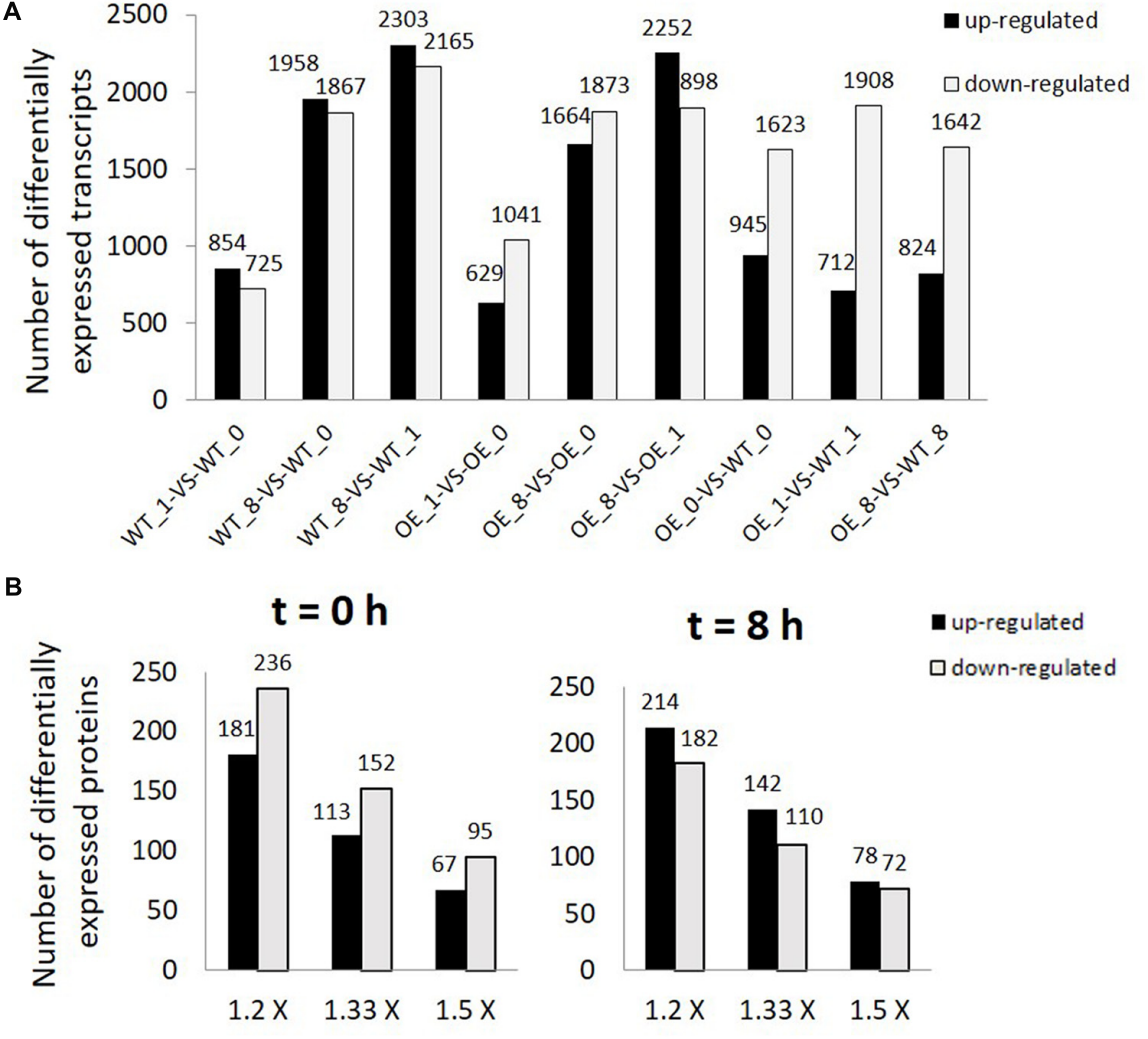
**Differentially expressed transcripts and proteins in overexpression (OE) line compared with WT.** **(A)** Number of differentially expressed transcripts (FDR ≤ 0.001, *P* < 0.001, and FC > 2). WT_0, WT_1, WT_8, OE_0, OE_1, and OE_8 represent WT and OE samples collected at *t* = 0, 1, and 8 h, respectively. **(B)** Number of differentially expressed proteins (1.2×: 1.2 fold change, 1.33×: 1.33 fold change, and 1.5 × : 1.5 fold change) between OE and WT samples collected at *t* = 0 (left) and 8 h (right).

When transcripts were compared between the OE line and WT, there were more suppressed transcripts than up-regulated transcripts in the OE line in all three time points (**Figure 2A**). Transcripts differentially expressed in OE line compared with WT at *t* = 0, 1, and 8 h were listed in **Supplementary Table S6**. Most of the differentially expressed genes were nuclear-encoded except one chloroplast-encoded transcript and a few transcripts encoded by the mitochondrial genome (**Supplementary Table S6**). Genes encoded by the chloroplast and the mitochondrial genomes which have significantly different transcript levels between OE line and WT with log2 ratio ≥1 or ≤–1 and *P*-value < 0.001 were found to be either ORFs or ribosomal subunit genes (**Supplementary Table S7**).

**FIGURE 3:**
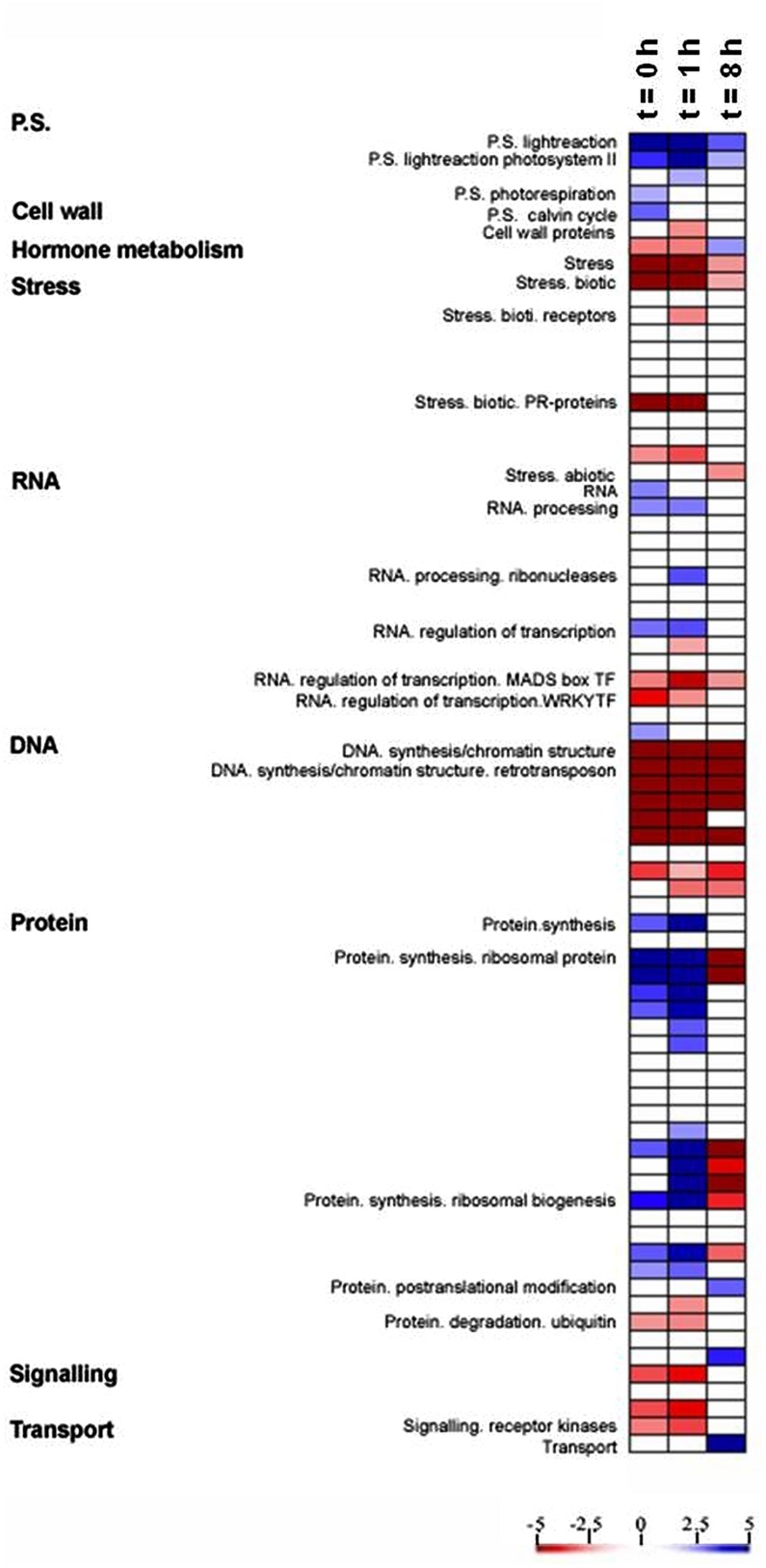
**Expression analysis in OE compared with WT.** A condensed PageMap display of altered pathways is shown. Gene expression data are presented as log_2_ fold changes in comparison with the same time point of WT. The analyzed points were *t* = 0, 1, and 8 h during light period. The data were subjected to a Wilcoxon test in PageMan, and the results are displayed in false-color code.

PageMan analysis (Usadel et al., 2006) was used to study the differential response to light between the OE line and WT by identifying significantly overrepresented functional groups (*P* < 0.01), using the ratio of the transcript level in the OE line to that in the WT for each time point and on the basis of Fisher’s exact and Wilcoxon tests for each category (**Figure 3**). This analysis facilitated the investigation of the global activation and/or repression of metabolic pathways and gene regulatory networks of the OE line in response to light. The OE line has been shown to exhibit higher electron transport rate (ETR) and non-photochemical quenching (NPQ) than the WT (Zhang et al., unpublished data). As would be anticipated, photosynthetic gene expression was up-regulated in the OE line in comparison to WT (**Figure 3**). However, a significant decline in photosynthetic gene expression across the light period was observed. Additionally, the expression of genes involved in photorespiration and the Calvin–Benson cycle were surprisingly only apparently up-regulated at the end of the night (*t* = 0 h) in the OE line (**Figure 3**). By contrast, genes associated to stress and redox responses were clearly down-regulated during light period in the OE line as compared to WT (**Figure 3**), although the expression pattern of the genotypes was more similar eight hours after the onset of illumination.

We additionally observed differential expression of transcripts involved in RNA processing and RNA transcription, which were up-regulated during end of night and beginning of light in OE line, but the transcription factor MADS box and WRKY families were down-regulated in the same time frame (**Figure 3**). It is also worth nothing that a strong down-regulation of transcripts involved in DNA synthesis and protein degradation were displayed in OE line during the light period while genes involved in the biosynthesis of ribosomal proteins were up regulated in the OE line (**Figure 3**), suggesting that the OE line may have a lower protein turnover rate, and the resources could be shifted to protein synthesis.

### Proteomics Studies and Differential Expressed Proteins

After strong cation-exchange (SCX) run, fractions were collected at every minute and finally 80 fractions were combined into twelve fractions for LC–MS/MS analysis. The profiles of SCX separation fractions are presented in **Supplementary Figure S1**. Spectrals, peptides, and proteins identifications were done by ProteinPilot software. Results of identified proteins, peptides and spectrals with different FDR threshold were presented in **Supplementary Tables S8** and **S9**. In total, 2,611 proteins, 24,135 peptides, and 156,907 spectrals were identified with 95% confidence in local FDR. Besides, when the global FDR was applied to the whole set of data, 2803 proteins, 25,521 peptides, and 167,625 spectrals were identified with 99% global FDR (**Supplementary Tables S8** and **S9**). Quality control measurements demonstrated that the protein data were confident for further study (**Supplementary Figure S2**). Our proteomic data identified 2,195 proteins with two or more peptides and with ProteinPilot unused score ≥1.3 (**Supplementary Table S10**). Differentially expressed proteins at both light (*t* = 8 h) and dark (*t* = 0 h) conditions were compared between the two lines (**Figure 2B**). In the dark, more proteins had lower abundances in OE line than in WT (181 up vs. 236 down, 1.2-fold change, *P* < 0.05). At *t* = 8 h, the protein abundances of 214 and 182 proteins were significantly up-regulated and down-regulated, respectively, in OE line versus WT (1.2-fold change, *P* < 0.05).

### Transcription and Translation of Chloroplast Genome

Among the 88 Coding Sequences (CDS) in the chloroplast genome, 87 CDS were detected in our transcriptome data at all three time points. The significant differentially expressed transcripts were selected with both 1.5- and 2-fold change (FDR ≤ 0.001) (**Supplementary Table S4**; **Supplementary Figure S3**).

At the end of the night, the abundances of 17 transcripts from the chloroplast genome were increased in the OE line but no transcripts were statistically suppressed. Eleven out of 17 upregulated transcripts encode ribosomal proteins. The transcripts of *TRNS.2*, *orf31*, *ycf3*, *petG*, *ndhF*, and *ndhH* were also increased in the OE line. The abundances of nine proteins were increased in the OE line and all of them encode ribosomal proteins, while the abundances of eight proteins were decreased in the OE line, including five photosystem II components, two ATP synthase subunits and one NDH complex component (**Supplementary Table S4**).

At 1 h after onset of illumination, only a few transcripts were up-regulated. Suprisingly, the transcripts of 16S and 23S ribosomal RNAs (*rrn16* and *rrn23*) were down-regulated in the OE line. By contrast, these ribosomal RNAs were up-regulated in the OE line after eight hours of illumination. At *t* = 8 h, only the transcripts of RNA polymerase beta’s subunit-2 (*rpoC2*), *psbD*, and *rps14* were significantly up-regulated in the OE line. Only four transcripts were suppressed, including two genes encoding PSII core proteins (*psbM* and *psbL*), *rpl20*, and *clpP1* encoding the caseinolytic protease P1. For protein abundance, the abundances of seven ribosomal proteins were increased in the OE line, while the protein abudances of three PS II components, two cytochrome b6f complex subunits and two NDH complex subunits were lower in the OE line (**Supplementary Table S4**).

By comparing the transcriptome and proteomic data between the end of the night and the middle of the day, the correlation of transcript and protein abundance is not high, except for a few ribosomal proteins (**Supplementary Table S4**). In general the OE line had a higher abundance of ribosomal proteins than the WT at both time points, and a higher abundance of 16S and 23S ribosomal RNAs than WT at the middle of the day, implying a higher translation rate in the OE line under illumination. Interestingly, a number of proteins involved in the linear photosynthetic electron transport chain, including components of photosystem II, NDH complex and cytochrome b6f complex, were suppressed in the OE line during the day. While in dark, when ATP is mainly produced in the mitochondria rather than in the chloroplast, the protein abundance of two chloroplastidial ATP synthase subunits were suppressed in the OE line.

The transcription of chloroplast genome is carried out by PEP (Plastid-Encoded Plastid RNA polymerase) and NEP (Nuclear-Encoded Plastid RNA polymerase). Previous studies showed that PEP transcribes a number of photosynthesis genes (*psaA*, *psbA-D, psbEFLJ*) under the control of six nuclear-encoded Sigma factors (Baba et al., 2004; Yagi and Shiina, 2014) and NEP transcribes a number of housekeeping genes (e.g., *accD, atpB, rpoB*) under the control of different NEP promoters (Yagi and Shiina, 2014). In addition, the transcription of some chloroplast genes (*atpA, clpP, rpl33, rrn5, rrn16*, and *rrn23*) are controlled by both PEP and NEP (Allison et al., 1996; Baba et al., 2004). Our transcriptome data showed that the transcript abundances of the above genes were not significantly different between the two lines, except for *rrn16* and *rrn23*. Hence, the transcription of these chloroplast genes by PEP and NEP is under complex regulatory control, and how availability of energy affects chloroplast genome regulation requires further studies.

### Transcription and Translation of Mitochondrial Genome

In total there are 122 mitochondria CDS genes in *Arabidopsis* according to TAIR 10 database^5^, of which 121 mitochondria CDS and 22 proteins with peptide number equal to or greater than two were detected in this study (**Supplementary Table S5**; **Supplementary Figure S4**). The transcript abundances were found to be significantly different between the OE and WT lines. At *t* = 0, 1, and 8 h, the abundances of 18, 8, and 13 transcripts were significantly higher in the OE line, respectively, whereas the abundances of 9, 19, 18 transcripts were significantly lower in the OE line, respectively. Hence, the transcription of the mitochondrial genome is greatly affected by the availability of energy. Comparing to WT, the transcript abundances of 5 genes (*cox1, ccb382, nad9, rps2*, and *orf131*) were consistently higher in the OE line at all three time points. For example, the transcript abundance of *cox1* increased from ∼1000 RPKM in WT to ∼2000 RPKM in the OE line at all three time points. In contrast, the transcript abundances of seven genes (*atp6-2* and 6 *orfs*) were consistently lower in the OE line at all three time points.

At the end of the night, most upregulated transcripts in the OE line encode hypothetical proteins. The transcripts of four NADH dehydrogenase components, two ATPase subunits, cytochrome c oxidase subunit 1, cytochrome c biogenesis of 382, and *rpl2* were also up-regulated in the OE line. In addition to the transcript of *atp6-2*, all other down-regulated transcripts encode hypothetical proteins. At *t* = 1 h, the transcript abundances of two NADH dehydrogenase, *cox1*, *rpl2*, *ccb382*, and *ccb203* were increased in OE line. In contrast, the transcript abundances of two NADH dehydrogenase subunits (*nad4l* and *nad3*), *atp6-2* and one ribosomal protein (*rps7*) were suppressed in OE Line (**Supplementary Table S5**). In the middle of day, the transcript abundances of *nad9*, *cox1*, *rpl2*, and *ccb382* were increased in OE line, while the transcript abundances of *atp6-2, ccb206*, and *rps5* were downregulated. Suprisingly, the abundance of 26S rRNA significantly increased in the OE line.

Transcription in eudicot mitochondria is carried out by two nuclear-encoded T3/7 phage RNA polymerase (RpoTm and RpoTmp; Liere et al., 2011). RpoTm is the basic RpoT for the transcription of most mitochondrial genes and RpoTmp plays a special role in the transcription of *cox1, ccmC, matR, nad1, nad2*, *nad6*, and *rps4* (Kuhn et al., 2009). Our RNA-seq showed that the transcript abundances *cox1* and *nad6* are higher in the OE line, whereas the transcript abundances of *matR, nad1, nad2*, and *rps4* were indifferent. Our data indicates that the transcription of mitochondrial genes through RpoTm and RpoTmp is under complex regulation.

Comparing to transcript abundance, not many proteins had significant changes in abundance. Amoug the 21 proteins detected in the proteomics analysis, only Nad2 and two ATP synthase subunits (Atp1 and Atpb) were upregulated and downregulated in the OE line, respectively, at *t* = 0 h (**Supplementary Table S5**). In the middle of day, protein abundances of the 21 proteins were not significantly different between the OE and WT lines.

### Transcription and Translation of Photosynthetic Complexes

**FIGURE 4:**
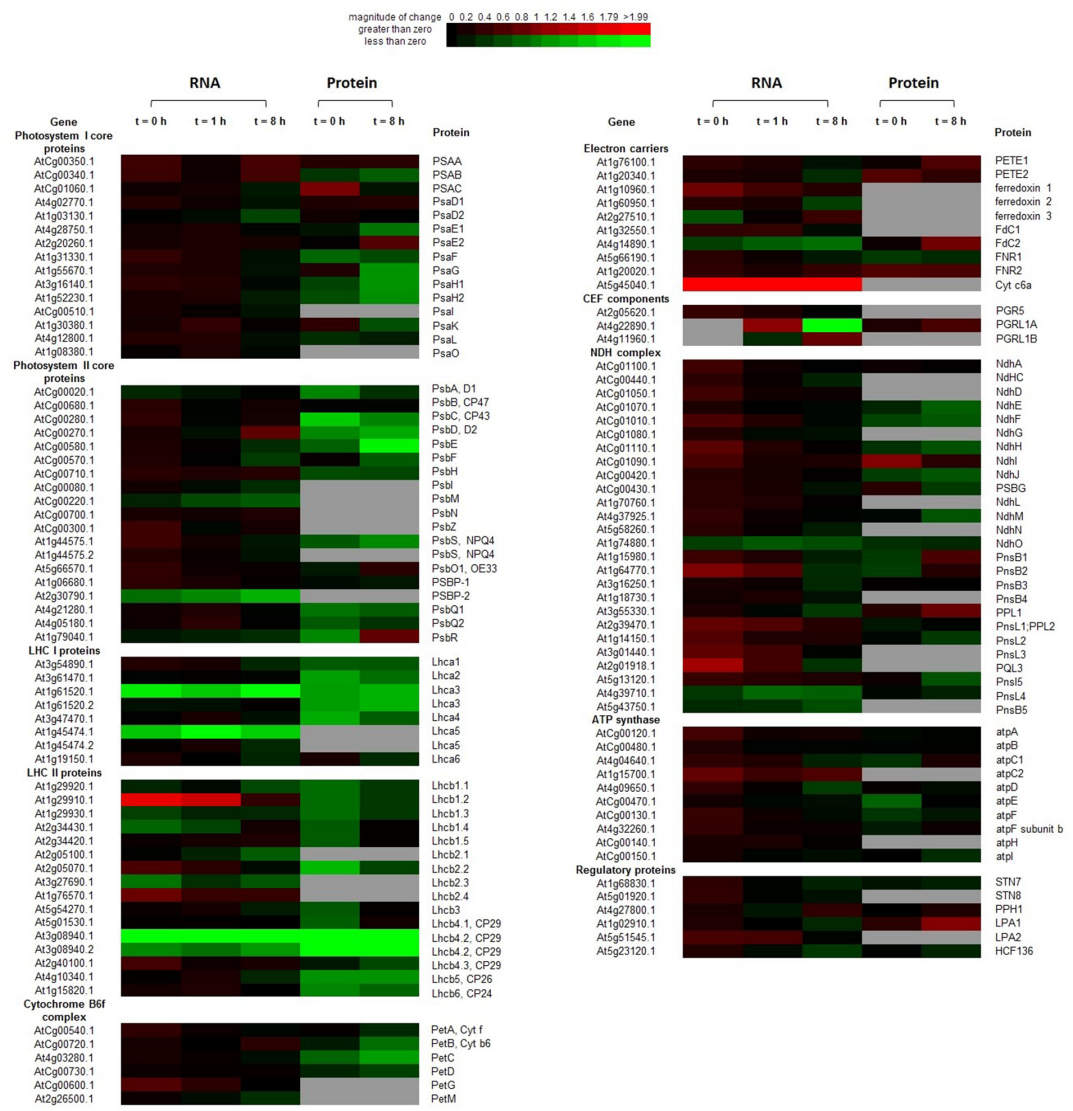
**Heatmap of transcription and translation profiles of photosystem compared WT with OE in 20-days-old leaves of *Arabidopsis*.** Each value was calculated by log_2_ ratio and colors were scaled per row with up-regulated in red and down-regulated in green. The gray ones indicated the missing data in RNA-Seq or iTRAQ. Heatmap was generated from http://bbc.botany.utoronto.ca/ntools/cgi-bin/ntools_heatmapper_plus.cgi.

Although mRNA levels of the core components of PSI and PSII, encoded by both the chloroplast and nuclear genomes, were not significantly changed during the dark to light transition in OE plants compared to WT, the transcription profiles of other components of the photosynthetic apparatus were significantly altered in the OE line (**Figure 4**; **Supplementary Table S11**).

At *t* = 0 h, transcripts encoding some LHCII components, soluble electron carriers (*PetG*, *ferredoxin 1*, and *Cytc6a*), multiple components of the NDH complex and ATP synthase (*atpC2*), were significantly up-regulated (*P* < 0.05) in the OE line. By contrast, transcriptions of some genes of the following complexes were suppressed in the OE line: one PSII gene, two LHCI genes, and three LHCII genes. In contrast to the mRNAs profiles, many proteins were suppressed in the OE line, including three PSI subunits, eight PSII subunits, three LHCI proteins, five LHCII proteins, PetC, NdhF and three ATP synthase subunits.

At *t* = 8 h, changes in genes expression profiles and protein abundances in the OE line were different with that of the end of night (*t* = 0 h). Transcription of *Cytc6a* was always up-regulated (*P* < 0.05) in the OE line in both dark and light conditions. *psbD* and *PGRL1B* were up regulated in the OE line. More transcripts were suppressed at *t* = 8 h, which include the several suppressed genes in dark (*Lhca3*, *Lhca5*, *Lhcb2.3*, *Lhcb4.2*, and *psbP2*). Transcriptions of *psbM*, *Lhcb2.1*, *Fd2*, *ndhO*, *pnsL4*, and *pnsL5* were suppressed in the OE line under light condition, which were not altered at the end of night. For proteins, the abundances of one electron carrier (FdC2), one CEF componets (PGRL1A), and one NDH complex components (PPL1) were increased (*P* < 0.05) in the OE line. The abundances of many proteins were decreased in the OE line. The suppressed proteins in PSI are docking sites for LHCII (PsaH1 and PsaH2), LHCI (PsaG and PsaK), and PETE (PsaE1). Besides, several components of PSII (psbC, psbD, and psbE), Cyb6f complex (PetB, PetC, and PetD), Lhca3, Lhcb4, Lhcb5, and NDH complex (NdhH, NdhJ, and NdhM) were also suppressed in the OE line (*P* < 0.05) (**Figure 4**; **Supplementary Table S11**). The mRNA levels of genes encoding several components of the photosynthetic complexes were validated by qRT-PCR (**Supplementary Figure S5**).

### Transcription and Translation of Key Metabolic Pathways

The transcriptional and translational profiles of enzymes involved in redox reactions and in central carbon metabolism, including the Calvin–Benson cycle, carbohydrate metabolism, glycolysis and the TCA cycle, were compared between the lines at both *t* = 0 h and *t* = 8 h (**Supplementary Tables S12**–**S15**).

We postulated that the mitochondria of OE line produce more ATP at the end of the night, which affects the transcription and translation profiles of plant cells. At the end of the dark period, the transcription of four genes involved in the Calvin–Benson cycle were up-regulated (one FBPase, one aldolase, one rubisco activase, and one transaldolase) and one was down-regulated (ribose-5-phosphate isomerase) in the OE line, whereas the abundances of three proteins [a fructose-bisphosphate (FBP) aldolase, a phosphoglycerate kinase, and a triose-phosphate isomerase] were decreased in the OE line (**Supplementary Table S12**). In carbohydrate metabolism, the transcript and protein abundances of several enzymes involved in starch metabolism, including -amylase, starch synthase, starch branching enzyme, and cytosolic starch phosphorylase were altered in the OE line (**Supplementary Table S13**), which suggests the energy status of plant affects starch metabolism significantly. In glycolysis, the transcription of almost all genes was unaltered between the lines, except for the downregulation of *PFK3* mRNA. Regarding proteins, the abundances of four enzymes were decreased in the OE line at *t* = 0, including a phosphofructokinase (PFK), a phosphohexose isomerase, a PK, and a triosephosphate isomerase (TPI) (**Supplementary Table S14**). In the TCA cycle, while only two genes were down-regulated in the OE line at transcriptional level, the protein abundances of an oxoglutarate dehydrogenase and a component of the succinate dehydrogenase (SDH) complex were increased. The protein abundances of a citrate synthase (CS), an aconitase (ACO), and a furmarase (FUM) were decreased in the OE line (**Supplementary Table S15**). For enzymes involved in redox reactions, there were larger differences between OE line and WT at the mRNA level compared to the protein level (**Supplementary Table S16**).

In the middle of the day (*t* = 8 h), when the output of carbon and energy from OE chloroplasts is higher (Sun et al., 2012), the transcript levels of the genes in the Calvin–Benson cycle were similar in OE line and WT with the exception of two genes down-regulated in the OE line. However, the protein abundances of sedoheptulose bisphosphatase (SBP) and two glyceraldehyde 3-phosphate dehydrogenase subunits (GADPH-A2 and GADPH-B) were significantly higher in the OE line, which could contribute to a higher rate of carbon assimilation (**Supplementary Table S12**). In carbohydrate metabolism, many enzymes involved in starch, sucrose and cell wall metabolism had higher protein abundances in the OE line (**Supplementary Table S13**). For glycolysis, there was almost no change at the transcriptional level, but five enzymes had lower abundances in the OE line (**Supplementary Table S14**). In the TCA cycle, no significant difference in protein abundances was found between OE line and WT, and only one gene (isocitrate dehydrogenase 1) was down-regulated in the OE line (*P* < 0.05; **Supplementary Table S15**). For redox proteins, the protein level of FdC1 was higher, but its mRNA level was lower in OE line at *t* = 8 h (**Supplementary Table S16**). The lack of correlation between mRNA and protein levels suggests that enzymes in central carbon metabolism are dominated by post-transcriptional regulation.

### The Transcription and Translation of the Respiratory Chain

In the respiratory electron transport chain in mitochondria, differential regulation of mRNA transcription was observed between the two lines at all three time points (**Supplementary Table S17**). The transcription of *GRIM-19* (Complex I component, involved in photorespiration), *Ndufs4* (Complex I) (Meyer et al., 2009), a 6 kDa peptide of F_0_ complex (At3g46430, Complex V) (Moghadam et al., 2012), and *ATP6-2* (Complex V) were consistently down-regulated in the OE line at all three time points. It should be noted that the transcription of *ATP6-2* in the WT were 131–141 RPKM but its level dropped to zero in the OE line in all three time points. By contrast, the transcription of *nad9*, *coxI* were consistently upregulated in the OE line at all three time points. For proteomics data, the protein abundances of NDC1 of alternative pathway, two subunits of Complex I (NAD2 and At2g20360), SDH2-2 of Complex II were higher in the OE line at *t* = 0 h. At the same time, the protein abundances of SDH5, a component of Complex III (MPPBETA), two subunits of ATP synthase (ATP1 and ATP5) were lower in the OE line. At *t* = 8 h, only the protein abundance of NDC1 was higher and that of two subunits of Complex I (24 kDa subunit and Ndufs4) and ATP5, were lower in the OE line (**Supplementary Table S17**).

### Impacts of AtPAP2 OE on Leaf Metabolite Levels

**FIGURE 5:**
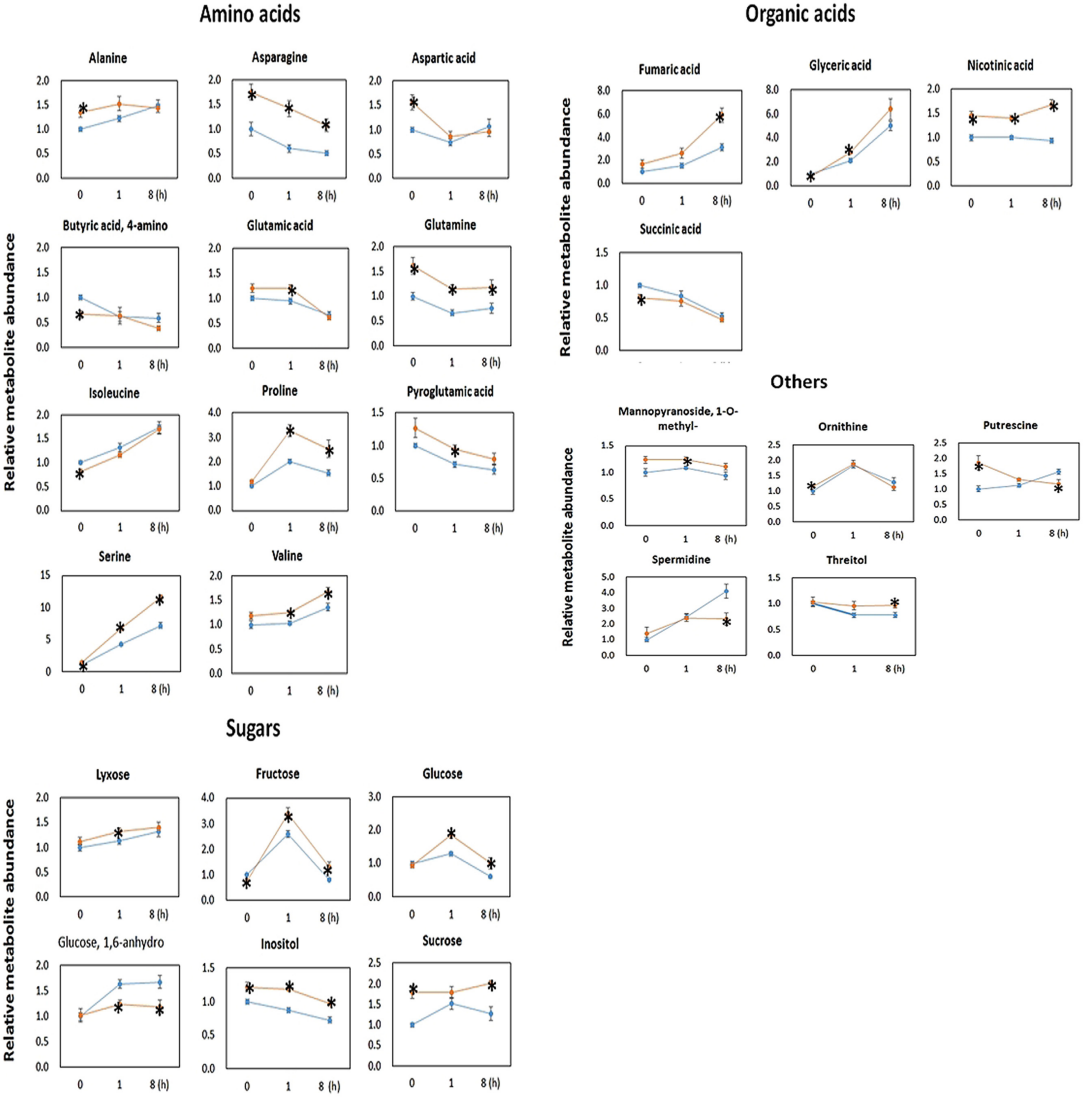
**Metabolomic data of 20-days-old *Arabidopsis* leaves collected at *t* = 0, 1, and 8 h after illumination.** Metabolites were measured through gas chromatography–mass spectrometry (GC–MS). The GC–MS data are normalized with the values obtained for ribitol (internal standard) and the FW used for the extraction (∼50 mg). Data are normalized to the mean response calculated for *t* = 0 h of WT. Values are presented as the mean ± SE of six biological determinations. Asterisks indicates values significantly different in OE line (orange) compared with WT line (blue) at each time point (*t* = 0, 1, and 8 h), respectively, as calculated by *t*-test (increase and decrease) with *P*-value < 0.05.

Metabolomics analysis showed that the OE line contained significantly higher level of sugars (**Figure 5**). The OE line contained significantly higher lyxose, fructose and glucose at *t* = 1 h, and significantly higher fructose, glucose and sucrose at *t* = 8 h. This finding is consistent with the findings of higher CO_2_ assimilation rates in transgenic *Camalina sativa* (Zhang et al., 2012) and potato overexpressing AtPAP2 (Zhang et al., 2014). After 8 h of darkness (*t* = 0 h), the OE line still contained significantly higher sucrose content but a lower level of fructose compared to WT. For metabolites in the TCA cycle, succinate was significantly lower in the OE line at *t* = 0 and fumarate was significantly higher in the OE line at *t* = 8 h.

The OE line contained significantly higher level of Asn, Gln, Ser, nicotinic acid, and inositol than WT at all three time points. In addition to Asn, Gln, and Ser, the levels of the following amino acids were significantly higher in the OE lines than WT at *t* = 0 h (Ala and Asp), *t* = 1 h (Glu, Pro, Val, and Pyroglutamic acid) and *t* = 8 h (Pro and Val) (**Figure 5**). From all investigated amino acids, only the levels of Ile and 4-aminobutyric acid (GABA) were significantly lower in the OE line at *t* = 0 h.

## Discussion

Consistent with this study, there are a wide range of observations that adenylates play a central role in the regulation of plant physiology (Geigenberger et al., 2010). Indeed, it is widely recognized that plants have different energy homeostasis than microbes and mammals (Saglio et al., 1983) as well as more complex compartmentation, which requires a more extensive transport system for adenylates (Haferkamp et al., 2011). Elevating the levels of adenylates via supply of adenine to potato tubers or adenosine to castor bean endosperm resulted in increases in starch biosynthesis and respiration and a general increase in anabolism, respectively (Loef et al., 2001; Florchinger et al., 2006). Similarly elevating the levels of adenylates by the repression of the plastidial adenylate kinase resulted in elevated starch biosynthesis in potato and enhanced growth in both potato and *Arabidopsis* (Regierer et al., 2002; Carrari et al., 2005). By contrast, a decrease in the adenylate energy state either by mutation of complex I of the respiratory chain in *Arabidopsis* resulted in suppression of germination and growth, and alteration in organic and amino acids (Meyer et al., 2009) whilst OE of apyrase led to a strong inhibition of starch biosynthesis, significant differences in the rate of respiration as well as considerable changes in gene expression (Riewe et al., 2008).

Despite considerable research effort, few comprehensive systems-based approaches have been performed to evaluate the effect of altered adenylate pools in plants. OE of AtPAP2 results in an elevated ATP content under both dark and illuminated conditions, which accounts for the OE plants high-energy status. AtPAP2 is dually targeted to the outer membranes of chloroplasts and mitochondria (Sun et al., 2012; Zhang et al., 2012). AtPAP2 selectively interacts with some components of the photosystems and plays a role in their import into chloroplasts (Zhang et al., unpublished data). It also interacts with a number of Multiple Organellar RNA Editing Factors (MORFs) and modulates the import of MORF3 into mitochondria (Law et al., 2015). OE of AtPAP2 results in a change in the photosystem composition and thylakoid architecture, which could lead to a higher photosynthetic efficiency and sucrose supply in the OE line (Zhang et al., unpublished data). OE of AtPAP2 solely in mitochondria resulted in early senescence and low seed yield (Law et al., 2015). These phenotypes showed that AtPAP2 can alter the physiology of these two organelles by modulating protein import. Hence, when we interpret the proteomics data of these two organelles, cautions should be taken as the increases in protein abundances of some nuclear-encoded proteins could be directly due to the role of AtPAP2 in protein import. By contrast, the changes in transcription and translation of these two organellar genomes are not directly linked to protein import, and a comparison of the OE line and WT at the end of the night reflects the impact of higher ATP output from mitochondria on chloroplasts and plant physiology. Also, comparison of the OE line and WT following illumination reflects the impact of higher energy output from chloroplasts on mitochondria and plant physiology.

Illuminated leaves of the OE line have higher ATP levels as well as a higher ATP/NADPH ratios than WT at all three time points (**Figure 1**). There are two major sources of ATP in plant cells: (i) linear electron flow (LEF) and cyclic electron flow (CEF) in chloroplast under light; (ii) the catabolism of assimilated carbon via glycolysis in the cytosol and the chloroplast, and quantitatively more importantly, by the TCA cycle and the respiratory electron transport chain in the mitochondria (Cheung et al., 2014). Carbon compounds used to generate ATP at night are produced from CO_2_ fixation in chloroplast during the day, so ultimately the high-energy status of the OE line must be resulted from a higher photosynthetic efficiency. Under illuminated conditions, ATP can be produced by LEF and CEF in the chloroplast (DalCorso et al., 2008) or from the mitochondrial electron transport chain (Bailleul et al., 2015). We cannot exclude the possibility that AtPAP2 OE enhances ATP production from both organelles through cooperation. A recent study on diatoms showed that the mitochondrial electron transport chain could consume excess reductants channeled from chloroplasts to produce ATP, which in turn could be used for carbon fixation in the chloroplasts (Bailleul et al., 2015).

Pulse amplitude modulation (PAM) measurement under normal growth conditions revealed that LEF and NPQ are higher in the OE line than WT (Zhang et al., unpublished data). Higher CEF in the OE line is indirectly supported by evidence of higher NPQ, which is mainly triggered by thylakoid lumen acidification (Munekage et al., 2002). NPQ is believed to be arisen from CEF because the ΔpH that is generated from LEF is rapidly consumed by the Calvin Cycle (Heber and Walker, 1992; Joliot and Finazzi, 2010). Two pathways of CEF around PSI have been postulated: a PGRL1-dependent route (wherein electrons flow from PSI to Fd to PGRL1 to Cytb6f/PQ to PETE and finally to PSI) and a NDH-dependent pathway (wherein electrons flow from PSI to Fd to FNR to NADPH to the NDH complex to Cytb6f/PQ to PETE and finally to PSI; Peng and Shikanai, 2011; Hertle et al., 2013). Yeast 2-hybrid and BiFC experiments showed that AtPAP2 interacts with PsaE2, FTRA2, FTRB, PQL1, and PQL3; but not with PsaE1, Leaf FNR, LHCA1, LHCB3, LHCB5, PsbQ1, PsbQ2, and PQL2 proteins (Zhang et al., unpublished data). Recent studies have revealed that PQL3 is also required for NDH activity (Yabuta et al., 2010) and that PQL1 is required for the stabilization of the NDH complex (Suorsa et al., 2010). Our proteomics data shows that some components in CEF pathways are altered at 8 h following the onset of illumination (**Figure 4**). The abundances of PsaE2, PGRL1, and FdC2 were increased (*P* < 0.05) in the OE line. By contrast, some proteins in PSII including (PsbC, PsbD, PsbE, PsbF, PsbS, and PsbQ1) were decreased (*P* < 0.05), which implies a shift in electron flow from LEF to CEF. This may be one of the possibilities that contribute to a lower NADPH level in the OE line eight hours following the onset of illumination (**Figure 1**). Another possibility is that the mitochondria in the OE line might be more active in consuming reducing power from chloroplasts and produce extra ATP under illumination (Bailleul et al., 2015).

Flux-balance analysis predicted that chloroplast and mitochondrial ATP synthases contribute 82 and 18% of ATP synthesis during the light condition, respectively, and the contribution of mitochondrial ATP synthesis decreases as light intensity increases (Cheung et al., 2014). Under illumination, chloroplasts export 3C compounds to cytosol, which are mainly consumed for sucrose synthesis. If there is a high ATP generation in the chloroplasts and the mitochondria of the illuminated leaf, the increase in ATP content in the OE line would be anticipated to result in a higher rate of carbon fixation and sucrose synthesis, which is supported by the higher sucrose phosphate synthase (SPS) activity in the OE line (Zhang et al., 2012). This in turn could lead to faster growth rate and higher biomass accumulation in the OE line. The extra sucrose allows the OE line to have more sugars at night (*t* = 0 h) for ATP production. In the dark, photosynthesis ceases and there is little ATP production from the chloroplast compared to the mitochondria. The higher ATP level in the OE line may be produced from mitochondria where extra sucrose can be used to generate more ATP via sucrolysis and mitochondrial respiration. Therefore, by comparing the transcriptome and proteome of OE line with that of WT at the end of night (*t* = 0 h), when ATP production from chloroplast is negligible, our study provides valuable information on the impact of high ATP output from mitochondria on global transcriptome and proteome of *Arabidopsis* leaf in the dark. It should be noted that the concentrations of Gln and Asn were higher in the OE line than the WT at all three time points, reflecting previous observations that when there is an ample supply of carbon, assimilation of nitrogen is also enhanced (Lawlor, 2002).

Comparing the transcriptome and proteome of the OE line and WT both at the end of the night and eight hours after the onset of illumination, we observed that the correlation between the levels of mRNA expression and protein abundance was low, suggesting that post-transcriptional regulation plays an important role in plants. This is similar to the findings in yeast and mammalian cells (Beyer et al., 2004; Tian et al., 2004), and is consistent with previous observations made in plants (Fernie and Stitt, 2012), particularly with respect to transcripts of the organellar genomes (Kahlau and Bock, 2008). Given that proteins are more closely related to biological function, we focus our discussion here on the relationship between proteome and metabolome. We should also bear in mind that the activities of certain central metabolic enzymes are not always correlated with their protein abundances nor their transcript levels but are rather related to factors of enzyme kinetics (Obata et al., 2011) with many key metabolic enzymes being regulated post-translationally, such as by allosteric regulations (by for instance ATP, ADP, citrate), by protein phosphorylation or lysine acetylation (Finkemeier et al., 2011) or by reassembly of protein complexes (Obata et al., 2011).

**FIGURE 6:**
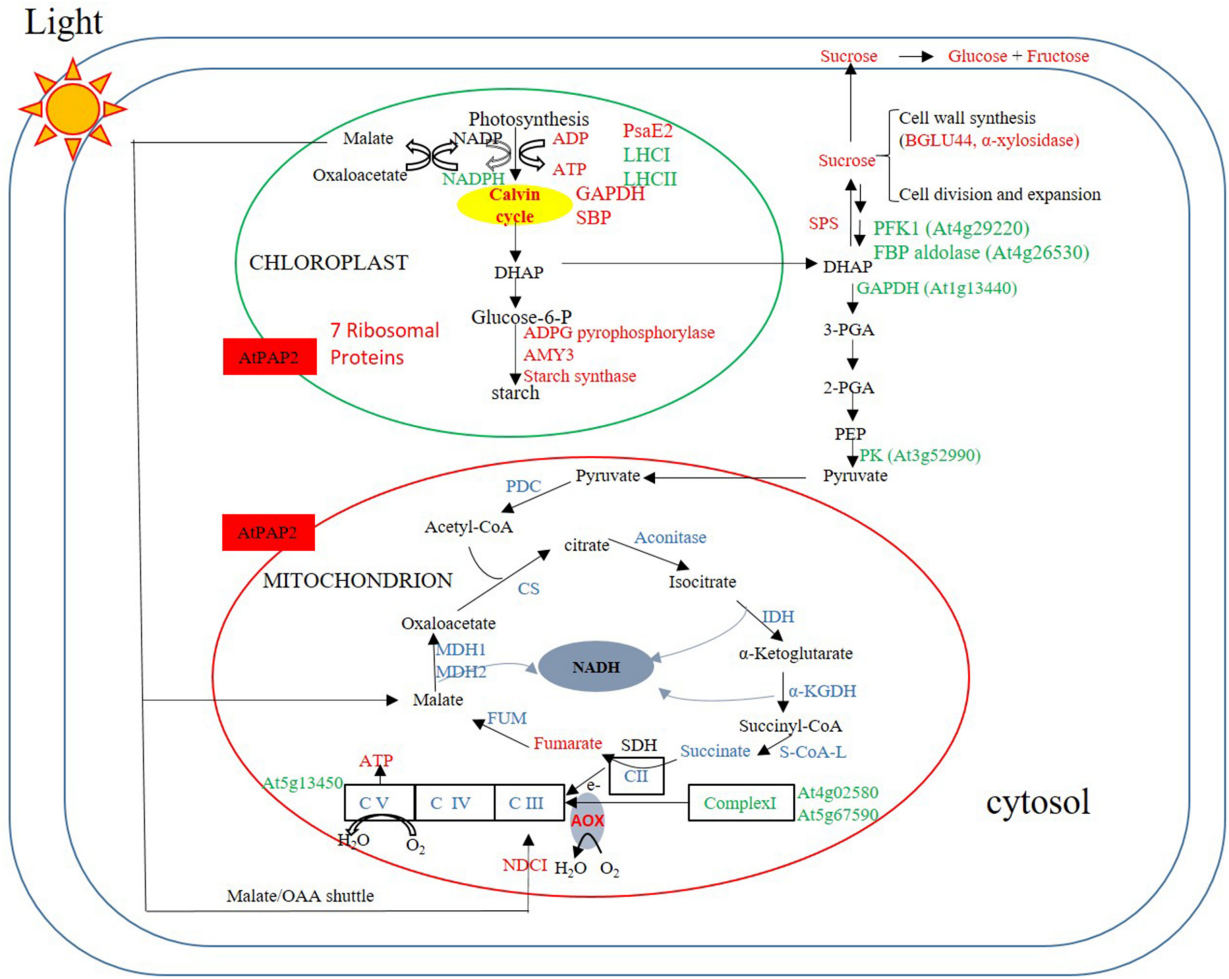
**Summary of protein and metabolites data at *t* = 8 h.** Up-regulated proteins and metabolites in the OE line (vs WT) are indicated in red and down-regulated proteins and metabolites are indicated in green color. Undetected proteins and metabolites are indicated in black color. Proteins and metabolites without significant changes are shown in blue color. Under illumination, the abundances of some enzymes in the Calvin–Benson cycle, starch synthesis and sucrose synthesis were higher in the OE line, whereas the abundances of some enzymes in the glycolysis pathway and TCA cycle were lower or unaltered in the OE line. Regarding the respiratory complexes, some Complex I subunits exhibited lower protein abundances, but that of Complex II–V were generally unaltered.

The comparison between the metabolome of the OE line and WT plants eight hours after the onset of illumination (**Figure 6**) revealed that the OE line has higher ATP and sucrose contents. These high energy molecules should ultimately be derived from a higher rate of photosynthesis and carbon fixation in the chloroplasts during the day. This is supported by the proteomics data which showed that the amount of photosystem PsaE2, proton gradient regulation 1 (PGRL1), and ferredoxin C2 (FdC2) proteins of the photosynthetic complexes, and glyceraldehyde 3-phosphate dehydrogenase (GAPDH) and SBP of the Calvin–Benson cycle were up-regulated in OE line in the middle of day. The higher carbon supply from chloroplasts thus provides abundant substrates for sucrose synthesis. Our previously reported enzyme activity studies revealed that the OE line exhibited higher SPS activities than the WT (Zhang et al., 2012), whilst higher sucrose production would be anticipated to promote growth activities such as protein and cell wall syntheses. In *Arabidopsis*, the chloroplast protein synthesis rate is threefold higher in the light than in the dark (Ishihara et al., 2015) whilst the sucrose level has been shown to correlate with polysome loading (Pal et al., 2013). The OE line studied here contain higher sucrose levels in both light and dark conditions so we anticipate that protein synthesis rates are higher than the WT in both conditions. Our proteomics data showed that the protein abundances of seven and nine chloroplast ribosomal proteins were higher in the OE line than WT under both light and dark conditions (**Supplementary Table S2**). In addition, the transcript abundances of chloroplast ribosomal 23S and 16S RNA are both twofold higher in the OE line than in the WT at *t* = 8h. These data support a higher protein synthesis rate in the OE chloroplast in both day and night times.

In an illuminated leaf, the photosynthetic light reaction is the major source of ATP, and other ATP producing processes such as glycolysis and the TCA cycle are inhibited. Consistent with this we observed a significantly lower protein levels for several glycolytic enzymes including PFK1, a FBP aldolase, a cytosolic glyceraldehyde 3-phosphate dehydrogenase (GAPDH), and a PK in the OE line following 8 h of illumination. No enzymes of the TCA cycle and proteins of Complexes II, III, and IV had their protein abundances down-regulated, whereas the protein abundances of two components, the 24 kDa subunit and the 18 kDa subunits of Complex I (Peng et al., 2011) and the delta subunit of ATP synthase (Geisler et al., 2012) were down-regulated (**Supplementary Table S17**). NAD(P)H dehydrogenase C1 (NDC1) of the alternative electron transport pathway was the only mitochondrial protein that was up-regulated in the OE line. While the alternative pathway reduces the yield of ATP production from the oxidation of NADH (Rasmusson et al., 2008), the external NDC1 might readily convert the excess reducing power exported from chloroplasts to ATP molecules through Complexes III, IV, and V of the respiratory chain (**Figure 6**).

**FIGURE 7:**
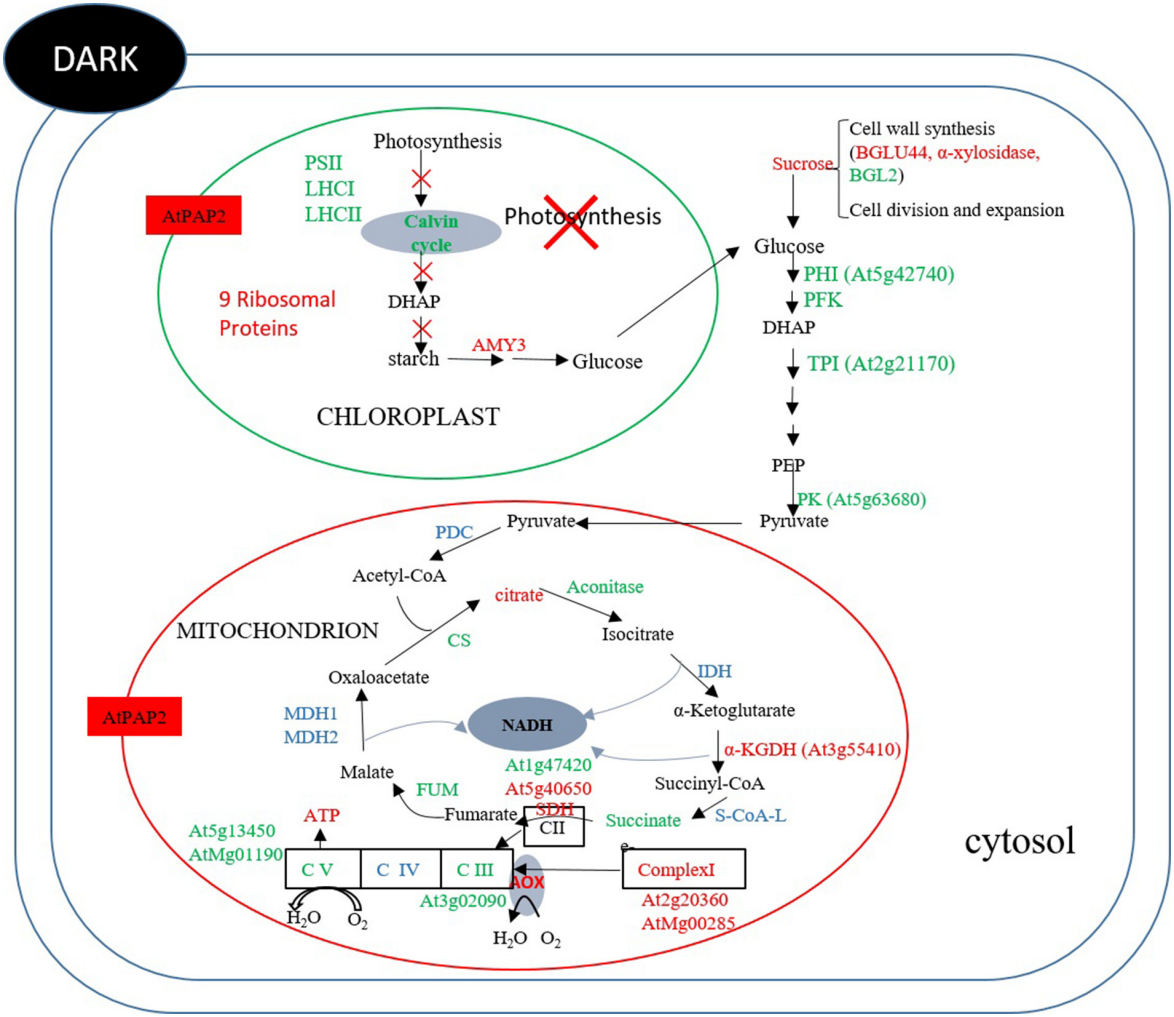
**Summary of protein and metabolites data at *t* = 0 h.** Up-regulated proteins and metabolites in the OE line (vs WT) are indicated in red and down-regulated proteins and metabolites are indicated in green color. Undetected proteins and metabolites are indicated in black color. Proteins and metabolites without significant changes are shown in blue color. In dark, the abundances of some enzymes in the Calvin–Benson cycle, starch synthesis and glycolysis were lower in the OE line, whereas the abundance of alpha-amylase for starch breakdown was higher in the OE line. Regarding TCA enzymes and respiratory complexes of mitochondria, the protein abundances of some enzymes and subunits were significantly different between the OE and WT lines, reflecting the complexity of mitochondrial physiology.

At the end of the night, when there is no photosynthesis, mitochondrion is the main organelle for ATP production. The OE line contained higher ATP and sucrose contents than WT. Comparing the protein abundance between OE and WT at the end of the night (**Figure 7**) revealed that two enzymes in the Calvin–Benson cycle, FBP aldoase and phosphoribulokinase (PRK), were lower, while the alpha-amylase-like (AMY3) starch degrading enzyme was higher in the OE line. For enzymes in the glycolytic pathway, the protein abundances of a PFK, a PK and a TPI were lower in the OE line. Compared to 8 h after illumination where none of the enzymes of the TCA cycle displayed significantly different protein abundances between OE line and WT, the protein abundances of CS, ACO3, and FUM2 were significantly decreased, whereas the protein abundances of SDH 2-2 and α-ketoglutarate dehydrogenase were increased in the OE line at the end of the night (**Supplementary Table S15**). Metabolome studies revealed that the level of succinate was significantly lower in the OE line, but the level of fumarate was higher (**Figure 5**), suggesting that the activity of the SDH (Complex II) might be higher in the OE line at the end of the night. Regarding the respiratory electron transport chain, the protein abundances of the 51 kDa protein of Complex I, SDH2-2 of Complex II, and NDC1 of the alternative respiratory pathway were higher, whereas NAD9 of Complex I, SDH5 of Complex II, and metalloendopeptidase of Complex III were lower in OE line at the end of the night. Two protein components of the mitochondrial ATP synthase, subunit 1 and the delta subunit (Geisler et al., 2012), were both decreaed (**Figure 7**). These changes in protein abundance revealed that the ATP production capacity of mitochondria is likely subject to complex regulation.

Chloroplasts and mitochondria are the major powerhouses of plant cells under light and dark conditions, respectively. The AtPAP2 OE line provides a good model to examine how higher energy output from chloroplasts affects the biology of mitochondria (**Figure 6**) and how higher energy output from mitochondria affects the biology of chloroplasts (**Figure 7**). In addition, this is also the first study that examined the impact of high energy supply on the global changes in the physiology of *Arabidopsis* in terms of gene expression, protein abundance, and metabolites levels.

## Author Contributions

BL designed the study. CL prepared the samples for RNA-seq, proteomics, and metabolite analyses and carried out leaf ADP, ATP, NADP+ and NADPH measurements, transcriptome analysis, and proteomics experiments. YZ carried out metabolite analysis under the guidance of AF. SC and SO provided expertises in bioinformatics analysis. YS carried out qRT-PCR. CL and BL wrote the manuscript. CC edited the manuscript.

## Conflict of Interest Statement

Boon L. Lim is the inventor of a US patent application (20130291224), which has been licensed to the Monsanto Company through his institution. The subject of the patent application is AtPAP2. The authors declare that the research was conducted in the absence of any commercial or financial relationships that could be construed as a potential conflict of interest.

## Footnotes

1 http://www.ncbi.nlm.nih.gov/geo/

2 http://www.arabidopsis.org

3 http://www.premierbiosoft.com/primerdesign/

4 http://www.arabidopsis.org/portals/genAnnotation/genome_snapshot.jsp

5 www.arabidopsis.org
