# Supplementary material for: Impacts of high ATP supply from chloroplasts and mitochondria on the leaf metabolism of Arabidopsis thaliana
Source: Front Plant Sci. 2015 Oct 28;6:922. doi: 10.3389/fpls.2015.00922 (PMC4623399; doi:10.3389/fpls.2015.00922)
Supplement: Supplementary file 1 [file Table_1.DOCX]

**Table S1 | List of primers used for qRT-PCR.**

| Primer Name | Primer sequences (5' to 3' direction) |
| --- | --- |
| PsaA_Forward | GGGCACAAGCATCTCAGGTAAT |
| PsaA_Reverse | AAGTTCTTGCCAATAACCACGC |
| PsaB_Forward | ACGGGTCAGTGGAATCTTTATG |
| PsaB_Reverse | ATCGGTTAGCCATAAACTTTGC |
| PQL1_Forward | TATCATCGCCGTGTAAACCAAC |
| PQL1_Reverse | CCTGTGCTAATGCTGATGACGT |
| PQL2_Forward | GGTCCTCTTCCAATTCCTCCTA |
| PQL2_Reverse | ATACATTCTTCCTTTCGTCCCG |
| ferredoxin 1_Forward | CTCTCTTCGGCCTCAAATCTTC |
| ferredoxin 1_Reverse | CATTCGACCTCTTGTTCTCCCT |
| Cyt c6a_Forward | GTTTCACCCATCTGTTTCCCTC |
| Cyt c6a_Reverse | CGTTGCACCAGGTTGAATAATG |
| FdC2_Forward | AATTCCTTCTCCTTCCCACTCC |
| FdC2_Reverse | AATTCAGTCGTCTTTCCGTCGT |
| Lhca3_Forward | GATTCCACCAGCAGGGACATAC |
| Lhca3_Reverse | GTACCAGTCCTGTAACCTCCGG |
| Lhcb2.3_Forward | TGCGTCGTACCGTCAAGTCTAC |
| Lhcb2.3_Reverse | GTGTCCCAACCGTAGTCTCCAG |
| Lhcb4.2_Forward | TGATCGGCTACATTGAGTTCCA |
| Lhcb4.2_Reverse | CGGGTCAGACGCTAGTCCTAAC |
| Actin2_Forward | CCCGCTATGTATGTCGC |
| Actin2_Reverse | AAGGTCAAGACGGAGGAT |
