# Supplementary material for: Impacts of high ATP supply from chloroplasts and mitochondria on the leaf metabolism of Arabidopsis thaliana
Source: Front Plant Sci. 2015 Oct 28;6:922. doi: 10.3389/fpls.2015.00922 (PMC4623399; doi:10.3389/fpls.2015.00922)
Supplement: Supplementary file 2 [file Table_2.DOCX]

**Table S2.**

A. Statistics of total number of sequencing reads mapped to Genes in TAIR 10.0.

|  | OE_0 | | OE_1 | | OE_8 | | WT_0 | | WT_1 | | WT_8 | |
| --- | --- | --- | --- | --- | --- | --- | --- | --- | --- | --- | --- | --- |
| Map to Gene | reads number | percentage | reads number | percentage | reads number | percentage | reads number | percentage | reads number | percentage | reads number | percentage |
| Total Reads | 65217952 | 100.00% | 64885466 | 100.00% | 67086798 | 100.00% | 65856848 | 100.00% | 68717410 | 100.00% | 66618050 | 100.00% |
| Total BasePairs | 5869615680 | 100.00% | 5839691940 | 100.00% | 6037811820 | 100.00% | 5927116320 | 100.00% | 6184566900 | 100.00% | 5995624500 | 100.00% |
| Total Mapped Reads | 49336837 | 75.65% | 51529284 | 79.42% | 51798246 | 77.21% | 50220939 | 76.26% | 54644882 | 79.52% | 52800526 | 79.26% |
| perfect match | 35412269 | 54.30% | 37068080 | 57.13% | 36637397 | 54.61% | 39421448 | 59.86% | 43404340 | 63.16% | 42190196 | 63.33% |
| <= 5bp mismatch | 13924568 | 21.35% | 14461204 | 22.29% | 15160849 | 22.60% | 10799491 | 16.40% | 11240542 | 16.36% | 10610330 | 15.93% |
| unique match | 38628227 | 59.23% | 40394837 | 62.26% | 41849875 | 62.38% | 40502486 | 61.50% | 43070199 | 62.68% | 42066874 | 63.15% |
| multi-position match | 10708610 | 16.42% | 11134447 | 17.16% | 9948371 | 14.83% | 9718453 | 14.76% | 11574683 | 16.84% | 10733652 | 16.11% |
| Total Unmapped Reads | 15881115 | 24.35% | 13356182 | 20.58% | 15288552 | 22.79% | 15635909 | 23.74% | 14072528 | 20.48% | 13817524 | 20.74% |

B. Statistics of total number of sequencing reads mapped to Genome in TAIR 10.0

|  | OE_0 | | OE_1 | | OE_8 | | WT_0 | | WT_1 | | WT_8 | |
| --- | --- | --- | --- | --- | --- | --- | --- | --- | --- | --- | --- | --- |
| Map to Genome | reads number | percentage | reads number | percentage | reads number | percentage | reads number | percentage | reads number | percentage | reads number | percentage |
| Total Reads | 65217952 | 100.00% | 64885466 | 100.00% | 67086798 | 100.00% | 65856848 | 100.00% | 68717410 | 100.00% | 66618050 | 100.00% |
| Total BasePairs | 5869615680 | 100.00% | 5839691940 | 100.00% | 6037811820 | 100.00% | 5927116320 | 100.00% | 6184566900 | 100.00% | 5995624500 | 100.00% |
| Total Mapped Reads | 54474405 | 83.53% | 56363893 | 86.87% | 56934770 | 84.87% | 56308614 | 85.50% | 59953853 | 87.25% | 58351588 | 87.59% |
| perfect match | 39211383 | 60.12% | 40656614 | 62.66% | 40408451 | 60.23% | 43870842 | 66.62% | 47185072 | 68.67% | 46153587 | 69.28% |
| <= 5bp mismatch | 15263022 | 23.40% | 15707279 | 24.21% | 16526319 | 24.63% | 12437772 | 18.89% | 12768781 | 18.58% | 12198001 | 18.31% |
| unique match | 51844143 | 79.49% | 53599596 | 82.61% | 54207466 | 80.80% | 53873689 | 81.80% | 57434680 | 83.58% | 55751167 | 83.69% |
| multi-position match | 2630262 | 4.03% | 2764297 | 4.26% | 2727304 | 4.07% | 2434925 | 3.70% | 2519173 | 3.67% | 2600421 | 3.90% |
| Total Unmapped Reads | 10743547 | 16.47% | 8521573 | 13.13% | 10152028 | 15.13% | 9548234 | 14.50% | 8763557 | 12.75% | 8266462 | 12.41% |

OE_0, OE_1, OE_8, WT_0, WT_1 and WT_8 represents RNA samples of OE and WT lines collected at t = 0, 1 and 8 h, respectively.
