# Supplementary material for: Impacts of high ATP supply from chloroplasts and mitochondria on the leaf metabolism of Arabidopsis thaliana
Source: Front Plant Sci. 2015 Oct 28;6:922. doi: 10.3389/fpls.2015.00922 (PMC4623399; doi:10.3389/fpls.2015.00922)
Supplement: Supplementary file 5 [file Table_7.DOCX]

**Table S7.** Differentially expressed organellar transcripts

|  | **GeneID** | **log2 Ratio** | **P-value** | **Gene Model Type** | **Primary Gene Symbol** |
| --- | --- | --- | --- | --- | --- |
| **OE_8/OE_0** | ATMG00020.1 | 1.98 | 0 | ribosomal_RNA | RIBOSOMAL RNA26S (RRN26) |
|  | ATMG00030.1 | 1.43 | 0 | protein coding | (ORF107A) |
|  |  |  |  |  |  |
| **OE_8/OE_1** | ATMG00020.1 | 1.97 | 0 | ribosomal_RNA | RIBOSOMAL RNA26S (RRN26) |
| **OE_0/WT_0** |  |  |  |  |  |
|  | ATCG00905.1 | 1.05 | 6.93E-15 | protein coding | RIBOSOMAL PROTEIN S12C (RPS12C) |
|  | ATMG00040.1 | -11.56 | 1.86E-34 | protein coding | (ORF315) |
|  | ATMG00070.1 | 1.06 | 1.39E-243 | protein coding | NADH DEHYDROGENASE SUBUNIT 9 (NAD9) |
|  | ATMG00370.1 | 4.82 | 6.43E-15 | protein coding | (ORF199) |
| **OE_1/WT_1** | ATMG00560.1 | 1.08 | 3.83E-216 | protein coding | (RPL2) |
|  | ATMG00830.1 | 1.30 | 1.12E-05 | protein coding | CYTOCHROME C BIOGENESIS 382 (CCB382) |
|  | ATMG00910.1 | 9.78 | 1.37E-07 | protein coding | (ORF215A) |
|  | ATMG01050.1 | -3.52 | 9.70E-06 | protein coding | (ORF159) |
|  | ATMG01090.1 | -8.08 | 9.30E-161 | protein coding | (ORF262) |
|  | ATMG01170.1 | -8.34 | 0 | protein coding | (ATP6-2) |
|  | ATMG01220.1 | -1.44 | 3.78E-31 | protein coding | (ORF113) |
|  |  |  |  |  |  |
|  | ATMG00040.1 | -12.05 | 2.02E-50 | protein coding | (ORF315) |
|  | ATMG00370.1 | 3.71 | 8.42E-12 | protein coding | (ORF199) |
|  | ATMG00810.1 | -1.06 | 2.73E-07 | protein coding | (ORF240B) |
|  | ATMG00830.1 | 1.18 | 6.61E-05 | protein coding | CYTOCHROME C BIOGENESIS 382 (CCB382) |
| **OE_8/WT_8** | ATMG00910.1 | 9.96 | 6.18E-09 | protein coding | (ORF215A) |
|  | ATMG01090.1 | -14.19 | 1.88E-183 | protein coding | (ORF262) |
|  | ATMG01170.1 | -8.19 | 0 | protein coding | (ATP6-2) |
|  | ATMG01220.1 | -1.23 | 1.74E-27 | protein coding | (ORF113) |
|  |  |  |  |  |  |
|  | ATMG00020.1 | 1.96 | 0 | ribosomal_RNA | RIBOSOMAL RNA26S (RRN26) |
|  | ATMG00030.1 | 1.58 | 7.94E-49 | protein coding | (ORF107A) |
|  | ATMG00040.1 | -5.45 | 4.23E-24 | protein coding | (ORF315) |
|  | ATMG00370.1 | 4.24 | 2.29E-18 | protein coding | (ORF199) |
|  | ATMG00560.1 | 1.05 | 5.73E-219 | protein coding | (RPL2) |
|  | ATMG00810.1 | -1.10 | 1.94E-06 | protein coding | (ORF240B) |
|  | ATMG00890.1 | 2.81 | 1.02E-05 | protein coding | (ORF106D) |
|  | ATMG00910.1 | 9.20 | 1.46E-05 | protein coding | (ORF215A) |
|  | ATMG01030.1 | 3.18 | 0.0002116 | protein coding | (ORF106E) |
|  | ATMG01090.1 | -13.86 | 7.02E-149 | protein coding | (ORF262) |
|  | ATMG01170.1 | -8.15 | 0 | protein coding | (ATP6-2) |
|  | ATMG01220.1 | -1.21 | 4.30E-21 | protein coding | (ORF113) |
