# Supplementary material for: Impacts of high ATP supply from chloroplasts and mitochondria on the leaf metabolism of Arabidopsis thaliana
Source: Front Plant Sci. 2015 Oct 28;6:922. doi: 10.3389/fpls.2015.00922 (PMC4623399; doi:10.3389/fpls.2015.00922)
Supplement: Supplementary file 6 [file Table_8.DOCX]

**Table S8.** Identification of protein, peptides and spectral yield at different FDR thresholds by ProteinPilot

| **Data Level** | **FDR Type** | **FDR** | **ID yield percentage** |
| --- | --- | --- | --- |
| **Protein** | Local | 1% | 2,382 |
|  |  | **5%** | **2,611** |
|  |  | 10% | 2,702 |
|  | Global | **1%** | **2,803** |
|  |  | 5% | 3,093 |
|  |  | 10% | 3,338 |
| **Distinct peptide** | Local | 1% | 18,349 |
|  |  | **5%** | **24,135** |
|  |  | 10% | 26,797 |
|  | Global | **1%** | **25,521** |
|  |  | 5% | 32,847 |
|  |  | 10% | 37,511 |
| **Spectral** | Local | 1% | 124,983 |
|  |  | **5%** | **156,907** |
|  |  | 10% | 171,075 |
|  | Global | **1%** | **167,625** |
|  |  | 5% | 210,221 |
|  |  | 10% | 221,905 |

Note: Local FDR indicated the FDR of an individual protein, peptide or spectral and Global FDR represented the FDR applied to the entire set of proteins, peptides or spectrals. FDR and ID yield in bold were the criteria for data analysis.

**Table S9.** Correspondence between FDR Levels and ProteinPilot Reported Confidences

| **Data Level** | **FDR Type** | **FDR** | **ID yield percentage** |
| --- | --- | --- | --- |
| **Protein** | Local | 1% | 99.0% |
|  |  | **5%** | **97.4%** |
|  |  | 10% | 93.5% |
|  | Global | **1%** | **85.5%** |
|  |  | 5% | 48.7% |
|  |  | 10% | 27.6% |
| **Distinct peptide** | Local | 1% | 99.3% |
|  |  | **5%** | **92.0%** |
|  |  | 10% | 77.4% |
|  | Global | **1%** | **85.3%** |
|  |  | 5% | 38.6% |
|  |  | 10% | 20.8% |
| **Spectral** | Local | 1% | 95.0% |
|  |  | **5%** | **60.6%** |
|  |  | 10% | 42.3% |
|  | Global | **1%** | **46.5%** |
|  |  | 5% | 0.0% |
|  |  | 10% | 0.0% |

Note: FDR and ID yield in bold were the criteria for data analysis.
