# Supplementary material for: Impacts of high ATP supply from chloroplasts and mitochondria on the leaf metabolism of Arabidopsis thaliana
Source: Front Plant Sci. 2015 Oct 28;6:922. doi: 10.3389/fpls.2015.00922 (PMC4623399; doi:10.3389/fpls.2015.00922)
Supplement: Supplementary file 9 [file Image_1.PDF]

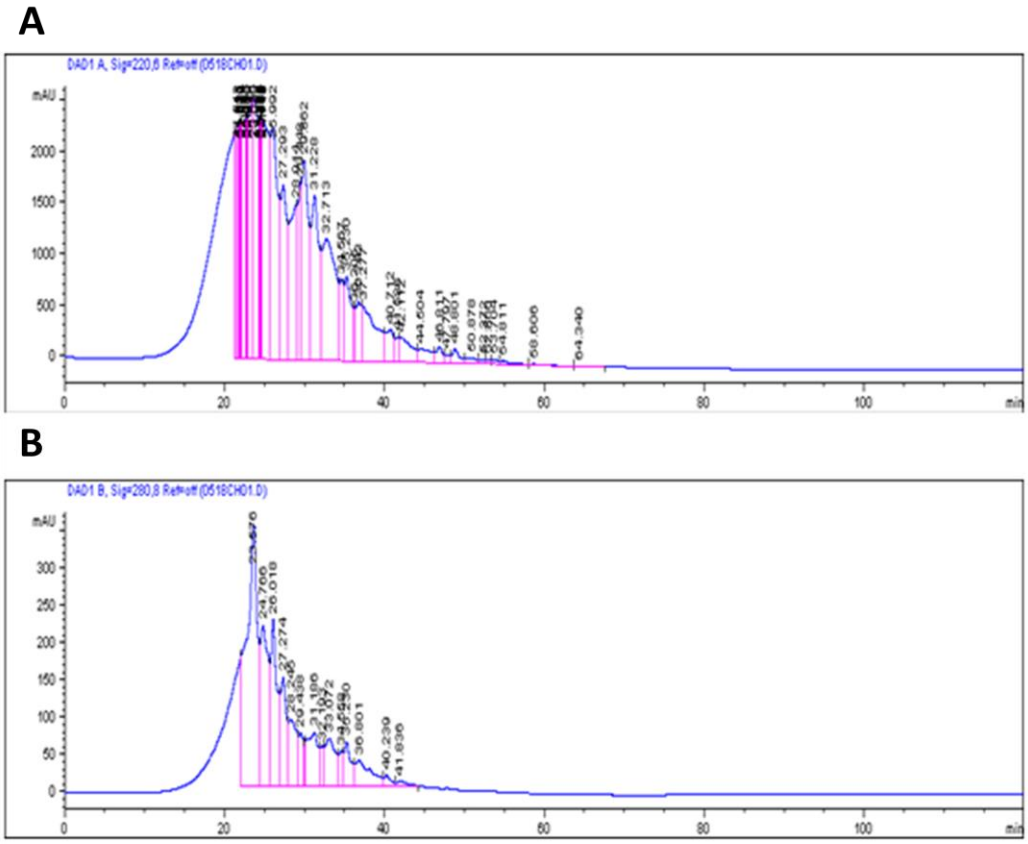

Figure S1 | Fractions of peptides by strong cation-exchange (SCX) chromatography.

(A) Profile of OD220; **(B)** profile of OD280.

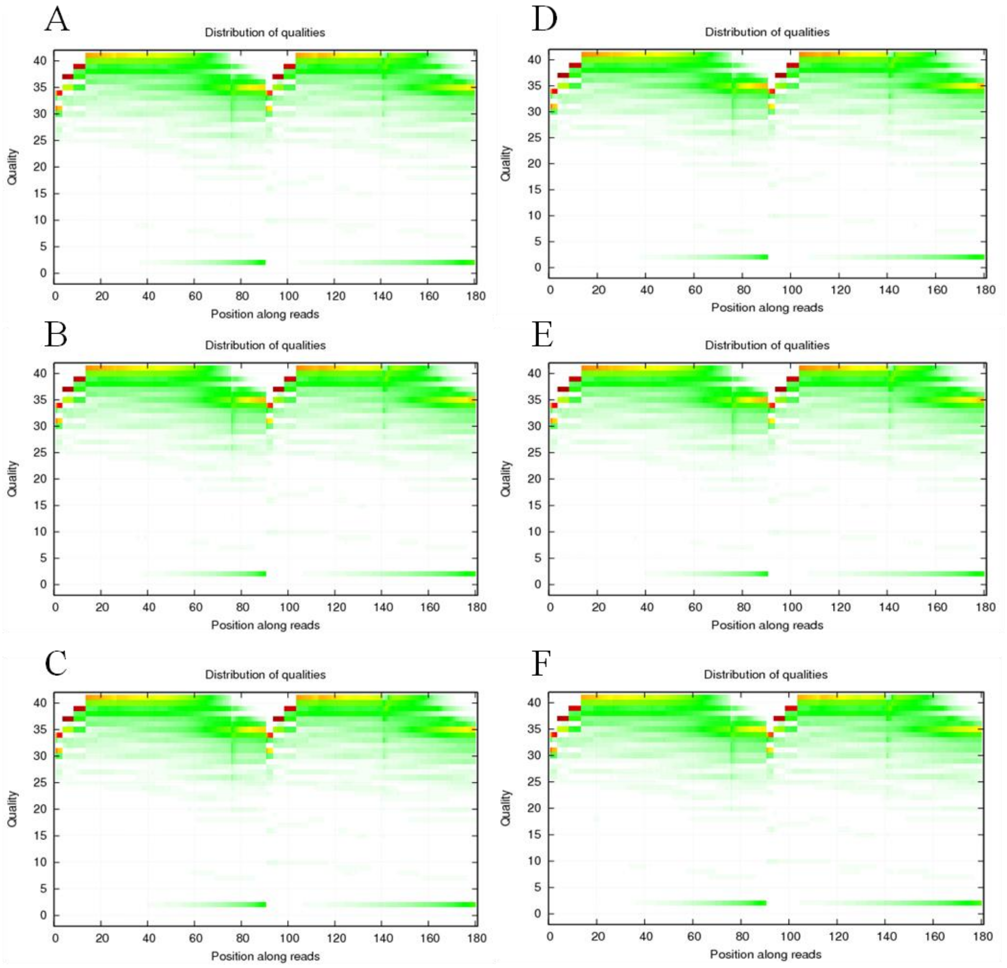

Figure S2 | Base sequences quality of the RNA-seq data. (A) WT_0 (t = 0 h); **(B)** WT_1 (t = 1 h); **(C)** WT_8 (t = 8 h); **(D)** OE_0 (t = 0 h); **(E)** OE_1 (t = 1 h). **(F)** OE_8 (t = 8 h). X-axis indicated positions along read, y-axis was quality value and each dot in the figure indicated the quality value along its corresponding position. All the values were above 20 which represented the QC of the samples was good.

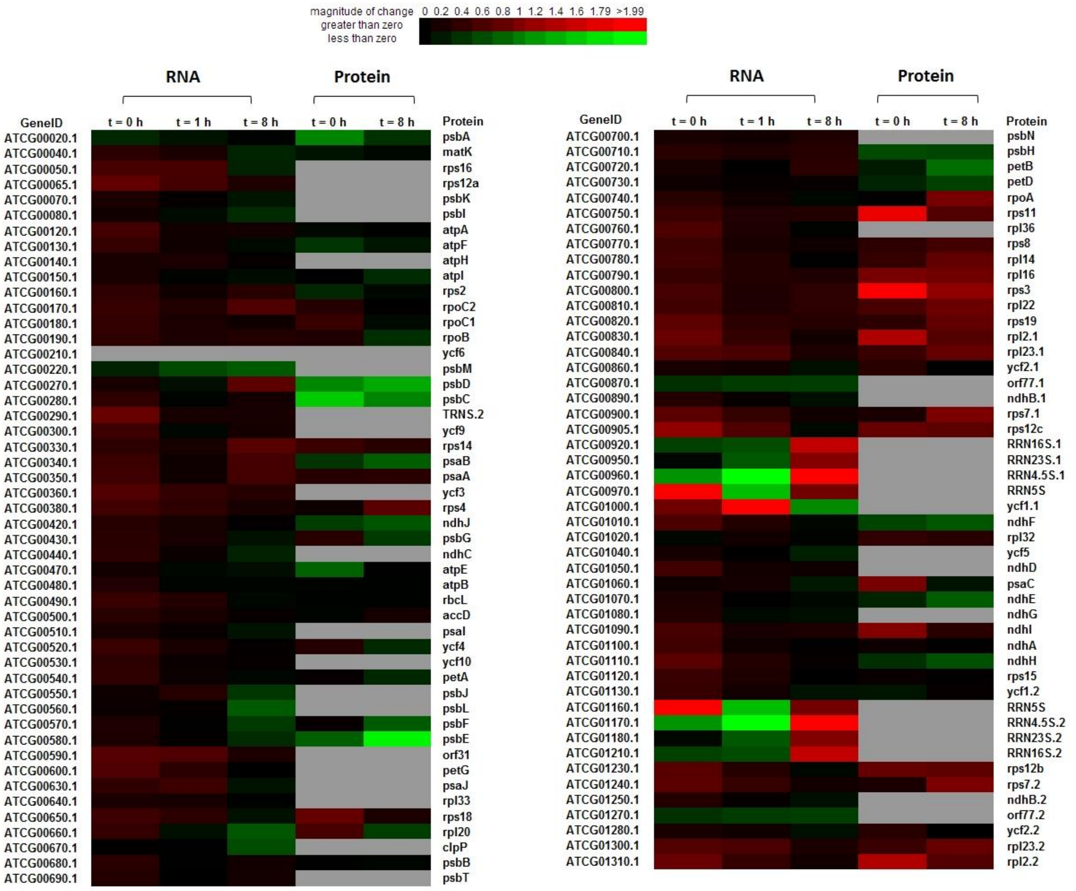

Figure S3 | Heatmap of chloroplast transcription and translation profiles compared WT with OE in 20-d-old leaves of Arabidopsis. Each value was calculated by log2 raito and colors were scaled per row with up-regulated in red and down-regulated in green. The grey ones indicated the missing data in RNA-Seq or iTRAQ. Heatmap was generated from http://bbc.botany.utoronto.ca/ntools/cgi-bin/ntools_heatmapper_plus.cgi.

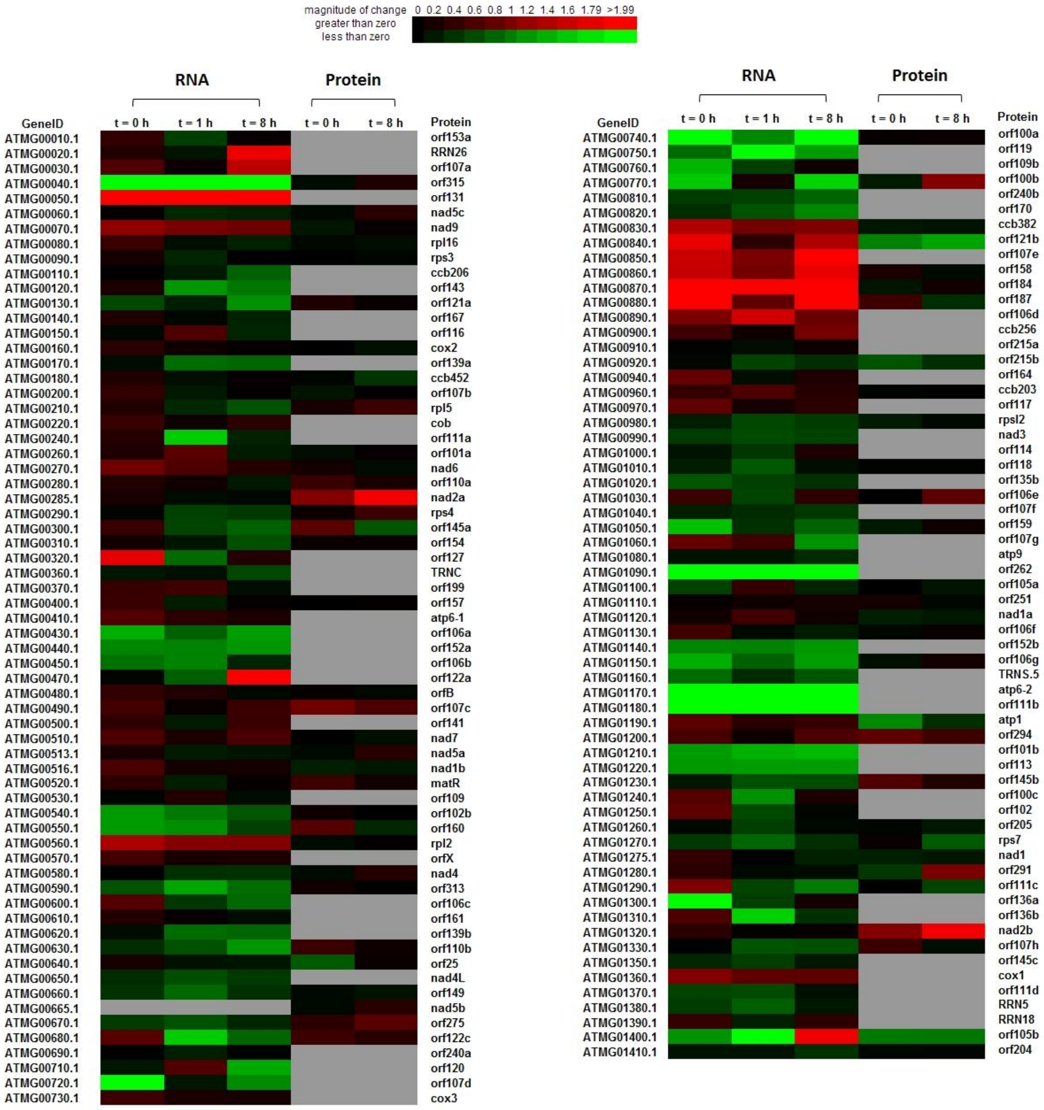

Figure S4 | Heatmap of mitochondrial transcription and translation profiles compared WT with OE in 20-d-old leaves of Arabidopsis. Each value was calculated by log2 raito and colors were scaled per row with up-regulated in red and down-regulated in green. The grey ones indicated the missing data in RNA-Seq or iTRAQ. Heatmap was generated from http://bbc.botany.utoronto.ca/ntools/cgi-bin/ntools_heatmapper_plus.cgi.

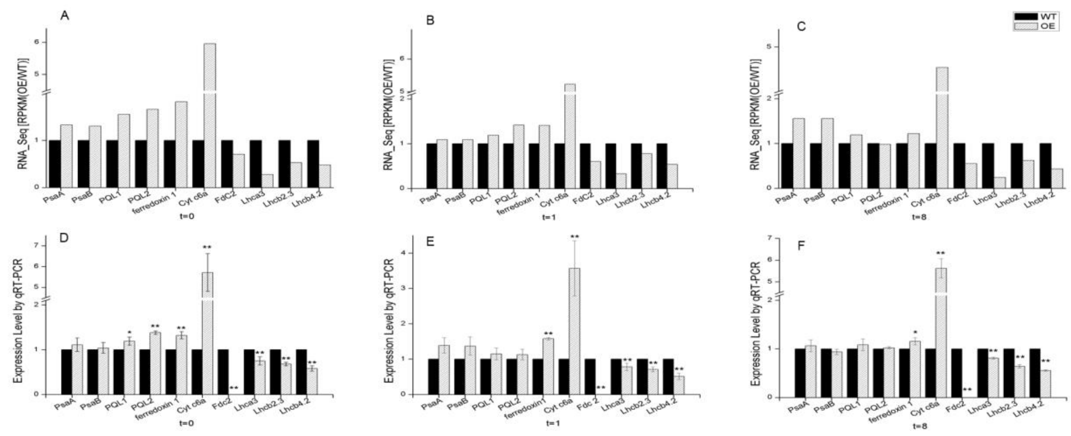

Supplementary Figure S5 | Validation the RNA-Seq results by qRT- PCR. The mRNA value in terms of RPKM detected in RNA_Seq were listed in at t = 0 (A), t = 1(B) and t = 8 h (C), respectively. The corresponding qRT-PCR validated results were shown at t = 0 (D), t = 1(E) and t = 8 h (F), respectively.
